# Understanding butanol tolerance and assimilation in *P* *seudomonas putida* BIRD‐1: an integrated omics approach

**DOI:** 10.1111/1751-7915.12328

**Published:** 2016-01-06

**Authors:** María del Sol Cuenca, Amalia Roca, Carlos Molina‐Santiago, Estrella Duque, Jean Armengaud, María R. Gómez‐Garcia, Juan L. Ramos

**Affiliations:** ^1^ Abengoa ResearchAbengoa, C/ Energía Solar 1, Palmas AltasSevilla41014Spain; ^2^ Bio‐Iliberis R&D. Polígono JuncarilC/ Capileira 7, PeligrosGranada18210Spain; ^3^ DSVIBiTec‐SSPILi2DLaboratory ‘Innovative Technologies for Detection and Diagnostics'CEABagnols‐sur‐CèzeF‐30200France

## Abstract

*P* *seudomonas putida* BIRD‐1 has the potential to be used for the industrial production of butanol due to its solvent tolerance and ability to metabolize low‐cost compounds. However, the strain has two major limitations: it assimilates butanol as sole carbon source and butanol concentrations above 1% (v/v) are toxic. With the aim of facilitating BIRD‐1 strain design for industrial use, a genome‐wide mini‐Tn*5* transposon mutant library was screened for clones exhibiting increased butanol sensitivity or deficiency in butanol assimilation. Twenty‐one mutants were selected that were affected in one or both of the processes. These mutants exhibited insertions in various genes, including those involved in the TCA cycle, fatty acid metabolism, transcription, cofactor synthesis and membrane integrity. An omics‐based analysis revealed key genes involved in the butanol response. Transcriptomic and proteomic studies were carried out to compare short and long‐term tolerance and assimilation traits. *P* *seudomonas putida* initiates various butanol assimilation pathways via alcohol and aldehyde dehydrogenases that channel the compound to central metabolism through the glyoxylate shunt pathway. Accordingly, isocitrate lyase – a key enzyme of the pathway – was the most abundant protein when butanol was used as the sole carbon source. Upregulation of two genes encoding proteins PPUBIRD1_2240 and PPUBIRD1_2241 (acyl‐CoA dehydrogenase and acyl‐CoA synthetase respectively) linked butanol assimilation with acyl‐CoA metabolism. Butanol tolerance was found to be primarily linked to classic solvent defense mechanisms, such as efflux pumps, membrane modifications and control of redox state. Our results also highlight the intensive energy requirements for butanol production and tolerance; thus, enhancing TCA cycle operation may represent a promising strategy for enhanced butanol production.

## Introduction

Currently ethanol constitutes 90% of all biofuels used; however, the sector offers a diverse range of promising alternatives. Other fuels, such as butanol have superior chemical properties: it has a higher energy content, lower volatility and corrosiveness for engines, and is compatible with existing fuel storage and distribution infrastructure. Thus, butanol has been proposed as the next‐generation biofuel to blend with gasoline, diesel and jet fuels (Dürre, 2011). Moreover, medium‐chain C4 alcohols can be produced from more sustainable feedstocks than biodiesel and can also be used as substitutes for existing chemical products such as a paint precursors, polymers and plastics. Its 2008, market value was estimated to be $5 billion (Cascone R., 2008).

Currently, the majority of butanol production is mediated by the petrochemical industry via propylene oxo‐synthesis using H_2_ and CO over a rhodium catalyst. Existing chemical butanol production costs are linked to the propylene market, which is extremely sensitive to the price of crude oil (Green, 2011). Butanol can also be produced by fermentation processes, employing anaerobic Gram‐positive bacteria, such as *Clostridium acetobutylicum*, through the acetone–butanol–ethanol (ABE) fermentation process at a ratio of 3:6:1 (Schiel‐Bengelsdorf *et al*., 2013). Several studies have pointed to the potential industrial interest of different *Clostridium* strains, such as *C. beijerinckii* BA101 and *C. acetobutylicum* P260, because they can use cheap feedstocks to drive fermentation and are considered to be second‐generation producers (Ezeji *et al*., 2007).The main limitations of ABE fermentation are related to the production of by‐products, the complex life cycle of Clostridia and its need to use strict anaerobic conditions.

To bypass the inherent limitations of Clostridia, efforts have been recently made to produce butanol using recombinant non‐native hosts, such as *Escherichia coli*, *Lactobacillus brevis, Bacillus subtilis*, *Geobacillus thermoglucosidasius*, *Saccharomyces cerevisiae* and *Pseudomonas putida*. The amount of butanol produced by these microbes ranged from 0.55 to 1.2 g L^−1^ (Atsumi *et al*., 2008; Steen *et al*., 2008; Nielsen *et al*., 2009; Berezina *et al*., 2010; Lin *et al*., 2014). These yields, while below those obtained with *Clostridium* (in the range of 10–20 g L^−1^), indicate the potential that these alternative platforms hold for industrial use. This is particularly true because cellular robustness is a major requirement for the microbial production of biofuel and biochemicals, as producer strains need to be resistant to the toxic solvents that are synthesized (Ramos *et al*., 2015).

While solvent tolerance is a relevant topic for these non‐native hosts, there is a scarcity of studies that explore the tolerance mechanisms within potential industrial strains. The best studied response to biofuels is that of *E. coli* to isobutanol. An isobutanol response network under aerobic conditions was mapped at the transcriptional level in *E. coli* using integrated data from gene expression, knockouts and principal component analyses (Brynildsen and Liao, 2009). It was proposed that under high isobutanol concentrations transcription factors ArcA, Fur and PhoB are activated as the result of altered membrane fluidity, the disturbance of electron flow and detection of quinone malfunctioning. The modification of gene transcription then leads to various alterations to central metabolism that involve the TCA cycle, respiration and metabolite transport (Rutherford *et al*., 2010). These studies suggest that the response to isobutanol tolerance is a complex phenotype that involves multiple mechanisms (Brynildsen and Liao, 2009; Rutherford *et al*., 2010).

*Pseudomonas putida* strains have efficient pump systems that are commonly used by microbes for detoxification purposes (Molina‐Santiago *et al*., 2014). These pumps are the basis for unusually high tolerance observed in some microbes towards a number of organic solvents and antibiotics. To investigate the potential of engineering better butanol producing hosts, we have performed an omics‐based study to elucidate the mechanisms involved in butanol tolerance and assimilation in *P. putida*. In this study, we used *P. putida* BIRD‐1, a metabolically versatile plant growth‐promoting rhizobacterium that is highly tolerant to desiccation (Matilla *et al*., 2011). *Pseudomonas putida* BIRD‐1 is highly capable at producing second‐generation biofuels using cheap carbon sources and has better short‐term tolerance to butanol than *P. putida* KT2440 and DOT‐T1E; in this article, we provide targets for improving this production by means of tolerance improvement and reducing assimilation of the target compound. Here, we present a global overview of strain selection, mutant library construction and transcriptomic and proteomic level studies within this context. Our findings reveal the multifactorial response that occurs in the presence of *n*‐butanol, which includes activation of efflux pumps and proteins related to oxidative stress, an increased demand of energy required to exclude butanol from the membranes and different modifications that enhance robustness of the strain.

## Results

### Selection of *P*. *putida* BIRD‐1 as a host for butanol production

A non‐native butanol producer should exhibit three relevant properties: tolerance to butanol, limited ability to assimilate butanol (to avoid its metabolization) and proficiency at using industrial carbon sources as feedstock for synthesis of butanol (i.e. glucose, lactate, succinate and glycerol). Because *P. putida* strains are highly tolerant to solvents (Ramos *et al*., 1997), we decided to explore the use of this strains. We tested three strains of *P. putida* whose genomes were known: DOT‐T1E (Ramos *et al*., 1995), KT2440 (Nakazawa, 2002) and BIRD‐1 (Matilla *et al*., 2011). The strains exhibited similar growth rates in M9 minimal medium using glucose, lactate and succinate (Table S1). *Pseudomonas putida* BIRD‐1 exhibited lower duplication rates in glycerol than KT2440 and DOT‐T1E. The three *P. putida* strains were able to assimilate butanol.

Regarding butanol tolerance, we performed different assays including growth tests in rich and minimal media in the presence of different butanol concentrations; we also determined survival rates after a sudden butanol shock. In M9 minimal medium with glucose as carbon source, BIRD‐1, KT2440 and DOT‐T1E grew with doubling times in the range of 1.46 to 1.93 h. In the presence of 0.8% (v/v) butanol, doubling times increased to 7.6 h, 15.3 h and 60.6 h for BIRD‐1, DOT‐T1E and KT2440 respectively. When cells were grown in rich medium [i.e. Luria–Bertani (LB)] and butanol, BIRD‐1 also doubled faster than the two other strains (Table S1). We carried out butanol shock experiments at different concentrations to estimate survival rates of the three *P. putida* strains used in this study. It should be noted that BIRD‐1 did not show any significant decrease in viability up to butanol concentrations of 2% (v/v), while at this concentration an acute decrease in viable cells was observed in KT2440, whereas DOT‐T1E showed intermediate cell viability (Fig. S1). These assays suggest that *P. putida* BIRD‐1 is able to withstand higher butanol concentrations than the other strains. Based on the high versatility for carbon source utilization, limited butanol consumption and higher tolerance to butanol, we choose to study the *P. putida* BIRD‐1 response to butanol in greater detail.

### Identification of genes involved in butanol tolerance and assimilation

**Figure 1:**
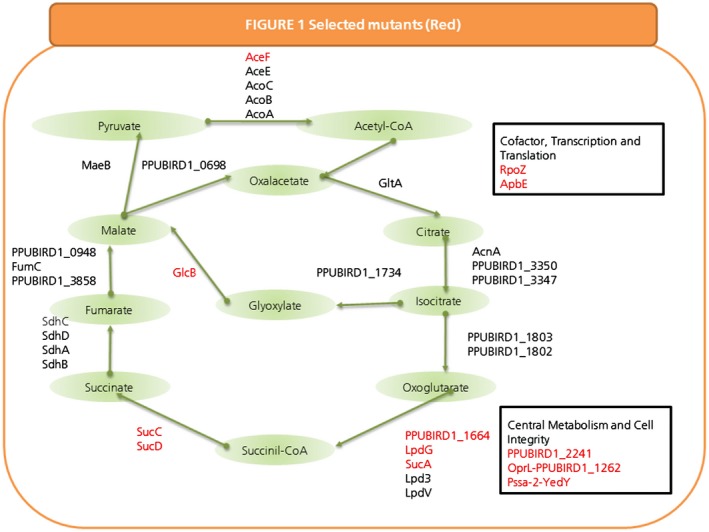
Schematic representation of *P*. *putida* BIRD‐1 mutants obtained after library screening using butanol as carbon source and/or stressor. Mutants affected after butanol exposure are presented. Mutants affected in assimilation or tolerance are shown in red. Several mutants are affected in TCA cycle and glyoxylate shunt pathways. Mutants affected in other processes are shown in black boxes.

We generated a *P. putida* BIRD‐1 mutant library containing a total of 7680 independent mini‐Tn*5* clones and carried out the selection assays described in the *Experimental procedures* section to identify key genes involved in tolerance and butanol assimilation. We identified 16 mutants (representing mutations in 14 distinct genes) that exhibited deficiencies in butanol tolerance, assimilation or both. Three of the mutants were compromised in butanol assimilation, three of them had defects in tolerance and 10 in assimilation and tolerance based on growth characteristics measured in a Bioscreen apparatus. The insertion point of the mini‐Tn*5* transposon in each of the mutants was mapped by means of arbitrary polymerase chain reaction (PCR) and Sanger sequencing as previously described (Caetano‐Anolles, 1993). The sequencing results showed that most of the mutants were affected in energy metabolism and conversion, coenzyme and nucleotide metabolism, and transport (Fig. 1, Table S2).

The three mutants that displayed compromised butanol assimilation had insertions at different locations within the gene encoding malate synthase G (GlcB), a key enzyme of glyoxylate pathway (energy metabolism and conversion). Solvent‐sensitive characteristics were observed in three mutants. The insertions interrupted genes related to energy generation and operation of TCA cycle. One of the mutants presented a transposon insertion in the *lpdG* gene, which encodes the dihydrolipoamide dehydrogenase E3 component of the branched‐chain α‐ketoglutarate dehydrogenase complex; while in the other two mutants, the mini‐Tn*5* was inserted at *sucA* and *sucD –* two genes that encode components of the thiamin‐requiring 2‐oxoglutarate dehydrogenase complex. These mutants are expected to be deficient in the generation of nicotine adenine dinucleotide (NADH) and to have limited ability to generate adenosine triphosphate (ATP) in respiratory chains, which would explain their sensitivity to butanol. Interestingly, 10 mutants were defective in butanol assimilation and at the same time were more sensitive to butanol than the parental BIRD‐1 strain. Three of these also presented insertions in TCA cycle‐related genes; namely, we found an insertion in PPUBIRD1_1664, which is a gene that is homologous to *kgdB* that encodes the E2 component of the branched‐chain α‐keto acid dehydrogenase. We also identified another mutant with an insertion in *sucC*, a gene that encodes a subunit of the succinyl‐CoA synthetase, which acts to convert succinyl‐CoA to succinate – a reaction that also involves the conversion of guanosine diphosphate (GDP) to guanosine triphosphate (GTP) and Coenzyme A with Sulfhydryl Functional Group (CoASH). It was also remarkable that one of the identified mutants had an insertion in the intergenic region between *lpdG* (as mentioned before, a gene that when mutated led to compromised butanol tolerance) and PPUBIRD1_1664, suggesting that the insertions exert a polar effect on the operon that interferes with the ability of the strain to assimilate butanol.

Two mutants had insertions in genes related to membrane stability. These included intergenic insertions between *pssa‐2*‐*yedY* and *oprL*‐PPUBIRD1_1262, which led to increased butanol sensitivity concomitant with compromised butanol assimilation. These genes encode proteins that are involved in lipid transport, metabolism and cell membrane stability. It should be noted that OprL is linked to cell membrane organization, and mutants in this gene have been previously described as being sensitive to various cellular stresses (38). One mutant had a mini‐transposon insertion in *apbE*, a gene that encodes a membrane‐associated lipoprotein involved in thiamine biosynthesis. Insertional mutants *aceF* (central metabolism) and PPUBIRD1_2241 (coenzyme metabolism) also exhibited altered butanol assimilation and tolerance.

Two of the mutants had defects in transcription and/or translation, and their deficiencies are likely due to alterations in overall metabolism (Llamas *et al*., 2003). An *rpo*Z mutant [ribonucleic acid (RNA) polymerase accessory protein] exhibited strongly impaired growth in the presence of the stressor and was unable to assimilate butanol as sole carbon source. This is likely due to the role of the RpoZ protein in RNA polymerase stability (Mukherjee *et al*., 1999; Mathew *et al*., 2005) along with potential polar effects on the gene encoding SpoT, which influences the cellular content of ppGpp alarmone (Gentry and Cashel, 1996). In addition, a single mutant in glutamyl‐Q tRNA (Asp) synthetase (*gluQ*, translation) was defective in butanol assimilation and tolerance due to its involvement in general metabolism.

### Transcriptomics

The transcriptomes of *P. putida* BIRD‐1 cells under four different physiological conditions were analysed by means of RNA‐seq. For comparative analysis, two independent biological replicates were carried out and four different conditions were tested: M9 with glucose was considered the control; M9 with butanol 0.5% as sole carbon source was used to elucidate expression changes involved in butanol assimilation; M9 with glucose and butanol 0.3% was used to study the long‐term tolerance response to butanol; and a shock of butanol was added to exponentially growing cells to study the short‐term solvent tolerance response. A total number of 34 267 239 reads were recorded, which represents average sequence mapping of 91.5% of the cases.

#### General overview

**Figure 2:**
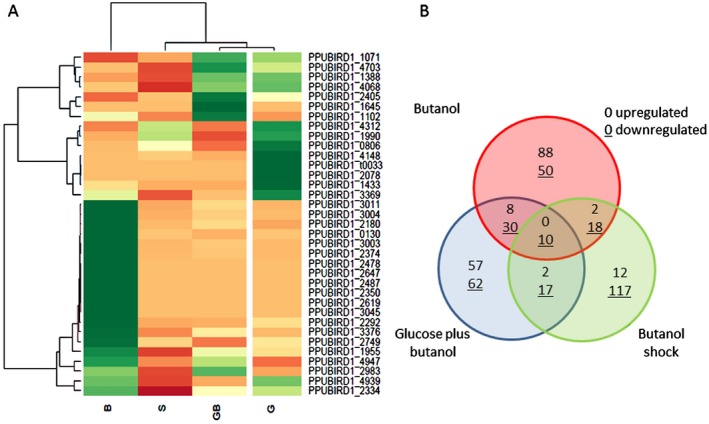
Transcriptomic analysis of *P*. *putida* BIRD‐1 after butanol exposure. A. Heat map and hierarchical cluster analysis of the most differentially expressed mRNAs in the presence of glucose; butanol; glucose and butanol; and butanol shock (*P*‐value < 0.05). Green represents mRNAs with high expression, and red indicates mRNAs with low expression. B. Venn Diagram of genes upregulated, downregulated or contra‐regulated among the three conditions, which are cells grown in butanol; cells grown in glucose and butanol; and cells recovered 1 h after 0.5% butanol shock.

After analysis of the expression profiles under four different growth conditions, the largest changes in expression patterns (upregulated and downregulated transcripts) were observed for the cells growing with butanol as the sole carbon source with respect the three other conditions (Fig. 2A).

Transcriptome analyses heat maps for each of the different growth conditions indicated that butanol assimilation requires deep metabolic changes. Cells growing with glucose plus butanol were most similar to control cells growing in glucose, although it should be noted that growth in the presence of butanol led to upregulation of a number of genes versus the control, which suggests co‐assimilation of substrates. For cells exposed to butanol shock, most of the transcripts were found to be downregulated with respect to the three other conditions. This is likely due to required readjustments to metabolism and the intensive expenditure of energy required to exclude the solvent, a situation similar to what has been observed in response to the addition of aromatic hydrocarbons to cultures of *P. putida* (Dominguez‐Cuevas *et al*., 2006).

To identify common and specific genes involved in metabolism and tolerance, a Venn diagram was generated (Fig. 2B). Transcriptomic analyses of cells grown in the presence of butanol and those grown with glucose plus butanol revealed that eight proteins were upregulated. One of these, known as *pcaL*, encodes the α‐subunit of β‐ketoadipate succinyl‐CoA transferase, which is involved in energy metabolism. This upregulated group also comprises a member of the GntR transcriptional regulator family of proteins, which are known to regulate membrane composition by changing the relative amount of saturated and unsaturated fatty acids. Other proteins in this group include: BioB (thiamine biosynthesis); a component of an ATPase (PPUBIRD1_1326); and several transcripts encoding hypothetical proteins.

A total number of 30 genes were found to be downregulated when cells were grown in butanol and glucose plus butanol. Examples of these include a gene that encodes the PilQ protein, which is involved in pili biosynthesis, and the *hmuV* gene, which encodes a hemin transporter. Other downregulated genes encoded transporters and secretion systems; an example of this is a gluconate transporter (PPUBIRD1_0697), a cation efflux protein (PPUBIRD1_1265) and a putative secretion system type IV protein (PPUBIRD1_4500). These findings indicate that in response to butanol, the cells conserve energy consumption through the tight control of efflux systems. As observed under all conditions, there were also altered levels of various hypothetical proteins (Table S3).

When we compared cells growing with glucose plus butanol to butanol shock, there were only two upregulated transcripts in common. Both of these encoded hypothetical proteins, namely, PPUBIRD1_1249 (homologous to FmdB, a regulatory protein with a zinc ribbon domain) and PPUBIRD1_1334 (a conserved hypothetical lipoprotein). These two proteins may play an important role in solvent defense mechanisms. Seventeen transcripts were found to be downregulated, including *flgH*, which is part of the flagellar ring complex, and *csrA*, a global regulatory protein that plays a role changing expression patterns in response to physiological stimuli. The downregulation of these genes indicate that the tolerance responses require the tight control of energy consumption and storage via a range of specific cell functions (such as motility) and more general mechanisms.

When cells were grown in butanol and glucose, upregulation of two biotin‐related transcripts that encode BioC and BioB proteins was observed. There are several key enzymes that require biotin; for example, the pyruvate carboxylase/oxaloacetate decarboxylase, which is involved in the TCA cycle, and others involved in lipid and fatty acid metabolism. In addition, biotin is important for fatty acid biosynthesis. The key role that biotin‐dependent genes plays in butanol solvent tolerance was previously described in *E. coli* by Reyes and colleagues (2011).

When cells were grown in butanol or were shocked with butanol, two commonly upregulated genes were detected. These are a short‐chain dehydrogenase (PPUBIRD1_1827) and a hypothetical lipoprotein (PPUBIRD1_2678), which may be involved in maintaining membrane stability. One gene was commonly downregulated – the *ftsL* gene, which is involved in cell division control.

The Venn diagram also reveals that for all three butanol conditions, only two transcripts were commonly downreglulated. These transcripts encoded transcriptional regulators; one that is a member of the TetR family of regulators (PPUBIRD1_2078) and another that is belonging to the AmrZ family of regulators (AlgZ, PPUBIRD1_1433). The TetR family of transcriptional regulators is known to be involved in the control of multi‐drug efflux pumps, catabolic pathways and adaptation to environmental conditions (Ramos *et al*., 2005). AmrZ regulators have been described to be involved in iron uptake as well as responses to environmental stimuli (Martinez‐Granero *et al*., 2014).

Regarding comparison of each condition and the control (Table S4), with cells grown with butanol as sole carbon source, 51% of the genes were found to be upregulated with respect to the control condition. Taking into account the genes that could be closely related to butanol uptake, upregulated genes included a component of an ABC transporter (PPUBIRD1_3000) that is an extracellular solute binding protein homologous to PedG, adjacent to the dehydrogenase‐PQQ dependent *qedH* gene (PPUBIRD1_3003), and a pentapeptide transcriptional regulator of the LuxR family (PPUBIRD1_3004). We also found upregulated genes for energy production, including: quinones and cytochromes (cytochrome c oxidase); isocitrate dehydrogenase (PPUBIRD1_1803) and other TCA‐related proteins, such as fumarate reductase (PPUBIRD1_3075). In addition, genes related with cellular division were primarily downregulated (i.e. FtsL, PPUBIRD1_4233).

When comparing cells grown in glucose plus butanol with the control, we found that 40% of the genes were upregulated. Remarkably, there was a strong upregulation of transcripts encoding the BkdR protein (PPUBIRD1_1442, 26). This protein is a regulator of branched‐chain α‐ketoacid dehydrogenase enzymes. Mutations in this gene led to a loss in the ability to use branched‐chain amino acids as carbon and energy sources (Madhusudhan *et al*., 1993). On the other hand, the most downregulated protein was the host specificity protein J (PPUBIRD1_2772).

We also analysed the fold change of transcripts under the butanol shock condition versus the control, for which 91% of total transcripts were downregulated. On the other hand, 9% of the transcripts were found to be upregulated, the highest upregulation was found to be the CyoD, a subunit of cytochrome oxidase (102‐fold).

### Proteomics

Three replicates were done of four different biological conditions: M9 with glucose (control); M9 with butanol 0.5% as sole carbon source (butanol assimilation); M9 with glucose and butanol 0.3% (long‐term tolerance response to butanol); and a butanol shock was added to exponentially growing cells (short‐term tolerance response to butanol).

The proteins associated with the soluble and insoluble material were extracted and analysed by high‐throughput tandem mass spectrometry as two separate fractions. The data set recorded from the 96 Liquid Chromatography‐ Mass Spectrometry/Mass Spectrometry (nanoLC‐MS/MS) runs comprised 707 041 MS/MS spectra. A total of 430 701 and 69 076 MS/MS spectra were assigned to peptide sequences for the soluble proteome and the insoluble‐associated proteins respectively. A total of 11 584 and 4243 different peptides were confidently listed respectively. Peptides validated the presence of 1086 and 591 proteins with at least two different peptides respectively. When considering the whole data set, a total of 1236 (without redundant) proteins were validated. Their relative quantities were estimated for each condition based on their respective spectral counts and normalized spectral abundance factors (NSAF).

**Figure 3:**
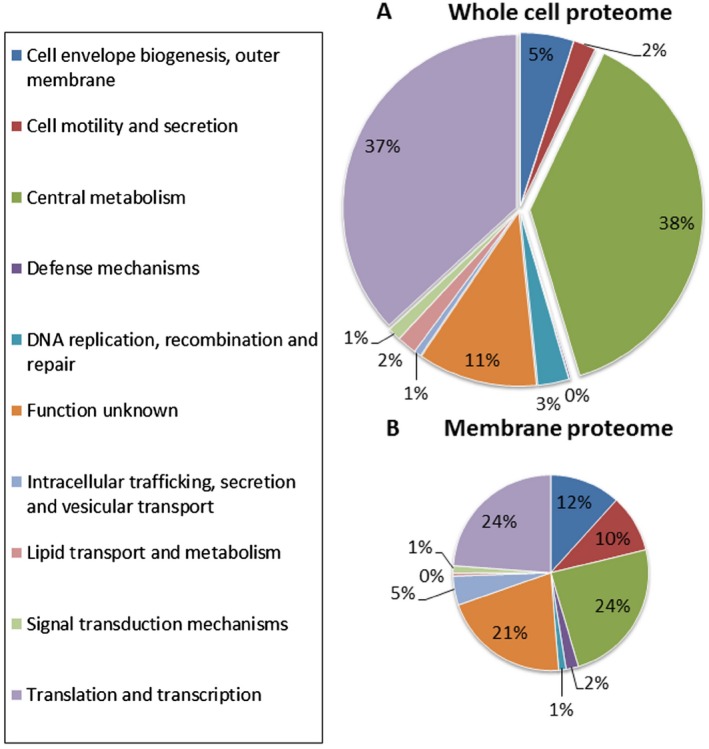
Proteomic analysis. Functional categories of genes displaying loss or gain in the following three conditions: cells grown in glucose and butanol; cells grown in butanol; and cells after sudden butanol shock. Relative quantity of proteins (NSAF) detected in (A) whole cell proteome and (B) membrane proteome are shown and are divided by functional categories.

Proteins involved in central metabolism, and translation and transcription were found to comprise 38% and 37% of total proteins (soluble and insoluble, respectively) in terms of quantities of the whole cell proteome when merging data from all four conditions. Proteins involved in biogenesis of the outer membrane represent 5% of the detected soluble proteins in terms of total MS/MS assigned. Figure 3A shows a general overview of the functional categories of the whole cell proteome, i.e. soluble and insoluble‐associated proteins weighted by the NSAF of the identified proteins in all conditions tested. The functional category results of the specific membrane‐associated proteins fraction are shown in Fig. 3B. In this case, 48% of NSAF are linked to central metabolism proteins, while translation and transcription related proteins account for 24%. As expected for such a specific proteome, proteins involved in cell envelope biogenesis (12%) and cell motility and secretion (10%) are more abundant in the membrane proteomes. Proteins involved in intracellular trafficking secretion and vesicular transport comprise 5% of the total protein quantities. For both proteomes, a relatively high amount of uncharacterized proteins (conserved hypothetical proteins) were detected. This global view of *P. putida* BIRD‐1 protein content indicates no specific bias in our proteomic strategy and points to central metabolism, and transcription and translation as key butanol‐related functional categories for systemic analysis.

Regarding butanol assimilation candidate proteins, we compared the control condition (C fractions) with cells grown in butanol as sole carbon source (B fraction) in terms of protein enrichment using the Tfold method of the patternlab program designed for label‐free shotgun proteomic data. The 1086 proteins from the whole‐cell proteome and the 591 proteins identified in the membrane‐associated proteomes were quantified and compared on the basis of their detection in at least two out of three replicates. The data are reported in supplementary Tables S5–S8. Using a TFold threshold above 2.5 and a stringent statistical level of confidence *(P* < 0.05), a list of 117 and 98 proteins were shown to be statistically more abundant in the B fraction compared with C fractions, while 92 and 72 proteins were less abundant in the whole cell proteome and membrane‐associated proteome respectively. Thus, the membrane‐associated proteome is more subjected to changes compared with the soluble proteome.

Most of the proteins that satisfied the *t*‐test and fold change cut‐off were related to central and lipid metabolism. The highest fold change, 278, was found for the acyl‐CoA dehydrogenase domain‐containing protein (PPUBIRD1_2240), followed by acyl‐CoA synthetase (PPUBIRD1_2241), which had a 245‐fold change. Both proteins are related to central carbon metabolism. The third highest fold change (148‐fold) was a β‐ketothiolase, which is involved in butanoate metabolism and central metabolism because it catalyses the conversion of acetyl‐CoA into acetoacetyl‐CoA. A protein that exhibited high abundance (as measured by NSAF) as well as a positive fold change was isocitrate lyase (PPUBIRD1_1734), a protein that is involved in central metabolism through its role in the glyoxylate shunt. In terms of abundance, the second most abundant protein was the histone family protein DNA‐binding protein HupB (45). The proteins LpdG, GlcB and SucA were also highly abundant, which suggests that these proteins are important for butanol metabolism. Regarding the quantity of downregulated proteins, a large number of them were involved in transcription and translation (i.e. Tuf‐2).

On the other hand, we found that porins and transporters, such as a sugar ABC transporter (PPUBIRD1_1065; –179), are primarily downregulated. The second most downregulated protein was PPUBIRD1_1059, a hypothetical protein that, according to a blast search, is an orthologue of glyceraldehyde 3‐phosphate dehydrogenase. In addition, genes involved in pentose phosphate pathways, such as Zwf, Edd and PgI (PPUBIRD1_1071, PPUBIRD1_1060 and PPUBIRD1_1073, respectively) were found to be strongly downregulated when butanol was used as sole carbon source.

#### Membrane proteome involved in butanol assimilation

QedH protein abundance was strongly upregulated (41.5‐fold change) in the membrane proteome and exhibited a NSAF of 4.76. QedH is a pyrroloquinoline quinone (PQQ)‐dependent alcohol dehydrogenase (QedH) located in the periplasmic space. Another highly upregulated protein, PPUBIRD1_0199, is an extracellular protein involved in surface adhesion (36). Porin B (similarly in the whole cell protein fraction) was sharply downregulated as well as the ATP‐binding subunit of the sugar ABC transporter. The most abundant non‐cytoplasmatic proteins were found to be SdhB (succinate deshydrogenae, subunit B) and a number of efflux pumps (i.e. TtgA of the TtgABC extrusion pump). In addition, we observed downregulation of the peptidoglycan‐associated lipoproteins OprL and OprF.

Focusing on long‐term response, in the glucose plus butanol condition, some of the upregulated proteins were the same as when butanol was used as sole carbon source condition. These include and acyl‐CoA dehydrogenase domain‐containing protein and acyl‐CoA synthetase (PPUBIRD1_2240 and 2241, respectively), suggesting that even when glucose is present, some butanol assimilation can occur simultaneously. Downregulated genes included IspB, a protein is involved in isoprenoid biosynthesis, and HlyD (PPUBIRD1_5002), a secretion family protein. In addition, a cyclic di‐GMP‐binding protein was strongly upregulated (13‐fold) in the membrane proteome.

#### Butanol tolerance

The butanol tolerance response of *P. putida* BIRD‐1 cells was studied for two modes: the long‐term response (glucose plus butanol condition) and the short‐term response (shock condition). However, some proteins were found in both conditions: 21 proteins were upregulated and 50 downregulated. We observed upregulation of MexF and ArpB (components of transporters), DnaK and OmpJ (chaperones), in addition to an aldehyde dehydrogenase (PPUBIRD1_0594); downregulated proteins included flagellin among others. After analysis of the membrane proteome, we also found that common upregulated proteins included efflux pumps (i.e. MexEF and TtgA and TtgB subunits).

For the short‐term response, we identified specific proteins with a high fold change in the whole cell proteome. These include ArpB (86‐fold), KatE (46‐fold), NdH (26‐fold) and the hypothetical protein PPUBIRD1_0113 (10‐fold). It should be noted that NdH is an oxidoreductase that controls proton translocation and KatE is a catalase; both proteins play a key role in oxidative stress defense.

### Genes and corresponding genes products upregulated and downregulated in proteomes and transcriptomes

A reduced number of transcripts and proteins were found to be upregulated or downregulated in both studies; however some of them are candidate targets for butanol tolerance or assimilation. Regarding short‐term tolerance, correlation between transcriptomics and proteomics data was analysed in order to ensure consistency. For the shock condition, LepA (a GTP‐binding protein), OprL and RplF (50S ribosomal protein) were downregulated. Importantly, it should be noted that the OprL mutant displayed significantly altered butanol tolerance and assimilation.

For the glucose plus butanol condition, CspA (cold shock protein), the electron transfer flavoprotein beta subunit and the hypothethical protein PPUBIRD1_4947 were upregulated in both experiments versus controls. RpoA (PPUBIRD1_0516, involved in transcription) and GlmU (PPUBIRD1_0057, involved in cell wall biogenesis) downregulation was also observed in both experiments for the glucose plus butanol condition versus controls.

Transcripts and proteins that were upregulated when butanol was the sole carbon source were RlmL, isocitrate dehydrogenase, QedH, CcoO, BdhA and also two hypothetical proteins (PPUBIRD1_2179 and PPUBIRD1_4947). Those that were consistently downregulated were KdsA (2‐dehydro‐3‐deoxyphosphooctonate aldolase), Pgm (phosphoglyceromutase), gluconate 2‐dehydrogenase and two hypothethical proteins (PPUBIRD1_5087 and PPUBIRD1_3386).

## Discussion

Harnessing the boundless natural diversity of biological functions for the industrial production of fuel holds many potential benefits. Inevitably, however, the native capabilities of any given organism must be modified to increase the productivity or efficiency of a bioprocess. From a broad perspective, the challenge is to understand sufficiently the mechanisms of cellular function such that one can predict and modify the microorganism. Butanol is one of the most promising alcohols for use as a biofuel and by the chemical industry, but production hurdles exist. In order to realize its potential, the butanol bio‐production process must achieve: increased conversion yields; efficient heterologous expression of the pathway in solvent tolerant strains, and; more versatile substrate compatibility (so that a greater variety of starting materials can be used). This study aims to explain the detailed cellular changes and responses that govern solvent tolerance and assimilation in a non‐native butanol producer, with the ultimate aim of advancing existing bio‐production methods.

### Existing setbacks and how to overcome low solvent tolerance

Low tolerance to alcohols by producer strains is one of the major challenges to industrial production. Short and medium‐chain aliphatic alcohols cause stress and lead to changes such as altered energy metabolism; altered saturated/unsaturated fatty acid ratios (which lead to altered membrane fluidity and efflux pumps function); expression of a number of stress proteins as heat shock proteins; altered cellular oxidation states, and; modification of the function of nutrient transporters (Papoutsakis and Alsaker, 2012).

*Pseudomonas putida* exhibits naturally high solvent tolerance (i.e. this microbe can survive in the presence of toxic chemicals such as trinitrotoluene (TNT), toluene and lineal and aromatic hydrocarbons) and a potent system for solvent detoxification, which is mediated by the expression of various membrane efflux pumps and by the ability to change the composition of membrane fatty acids (to help reduce membrane permeability) (Ramos *et al*., 2002; Udaondo *et al*., 2012). Other key determinants for solvent tolerance in *P. putida* include the ability to induce reactive oxygen species (ROS) scavengers and a number of chaperones for fast re‐folding of denatured proteins, and induction of the TCA cycle to ensure that there is sufficient energy to carry out these functions (Ramos *et al*., 2015). We tested several strains of *P. putida* as potential hosts for butanol production. While all of them showed the above properties, the BIRD‐1 strain was chosen as a host for future industrial scale‐up due to the ability to efficiently metabolize diverse starting substrates such as glycerol (as sole carbon source), glucose derived from lignocellulose and end products of the fermentation industry (i.e. lactate and succinate). BIRD‐1 grew faster than DOT‐T1E and KT2440 strains in the presence of butanol and it survived better after a sudden butanol shock, indicating that BIRD‐1 is the most robust of the strains in regard to butanol tolerance.

### The butanol assimilation pathway in *P*. *putida*

A previous study reported that in *P. butanovora*, butanol was assimilated via its conversion to butyraldehyde, and thereafter to butyrate (Arp, 1999). Furthermore it has been suggested that, after the action of several alcohol and aldehyde dehydrogenases, fatty acid oxidation enzymes may also be involved in butanol assimilation (Gulevich *et al*., 2012). Our current work revealed that a mini‐Tn*5* mutant deficient in the GlcB (a glyoxylate shunt pathway enzyme) is compromised for butanol assimilation. The importance of the glyoxylate shunt pathway to butanol assimilation was also supported via our proteomics studies, which showed that another glyoxylate shunt protein, isocitrate lyase, was upregulated when butanol was used as the sole carbon source. In addition, our proteomic analysis also detected high levels of an acyl CoA dehydrogenase domain containing protein (PPUBIRD1_2240). Taken together, these results identify the glyoxylate shunt as a key pathway that drives butanol to central metabolism.

**Figure 4:**
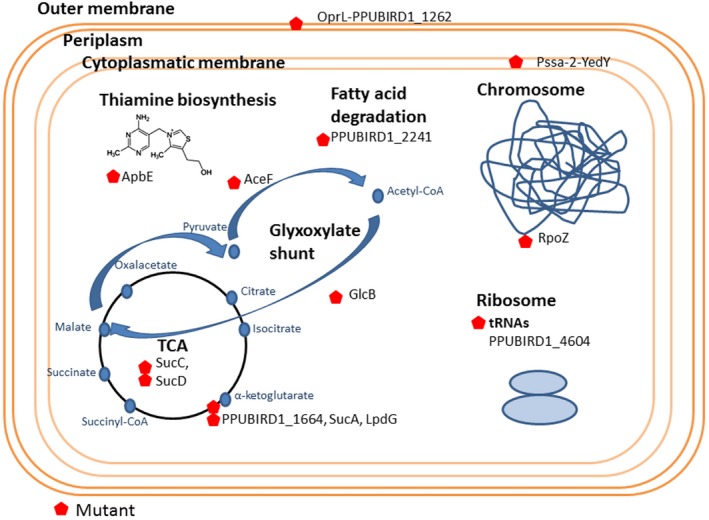
Butanol assimilation pathways. The putative butanol assimilation pathways are described. Butanol is assimilated via acetyl‐CoA and enters in central metabolism through the glyoxylate shunt. Candidate genes and fold changes in proteomic assays are shown.

The proteomic analysis indicated that in the initial steps of butanol assimilation, QedH and other aldehyde dehydrogenases (PPUBIRD1_0594, 2995, 5072, 2327) may be involved in conversion of butanol to butyraldehyde. Subsequently, butyraldehyde is likely converted into butyrate via the action of one or more aldehyde dehydrogenases (*i.e*., PPUBIRD1_2995 and/or PPUBIRD1_5072). Also, we found several candidate proteins that could catalyse the conversion of butyrate into butyryl‐CoA, and that an acyl‐CoA synthetase candidate was found to be induced 245‐fold (PPUBIRD1_2241). The gene encoding this acyl‐CoA synthetase is adjacent to a gene encoding an acyl‐CoA dehydrogenase domain‐containing protein (PPUBIRD1_2240), which is induced 278‐fold and that may serve to convert butyryl‐CoA to crotonyl‐CoA. Another part of this putative pathway may involve an upregulated enoyl‐CoA hydratase (PPUBIRD1_3766), which can convert crotonyl‐CoA to hydroxybutyryl‐CoA. Other candidates well represented in the proteome may be responsible for further conversions (PPUBIRD1_2007, PPUBIRD1_3518, PPUBIRD1_2008 and PPUBIRD1_4333). As stated above, the entry point to central metabolism likely occurs through the glyoxylate shunt. Further studies and experiments, such as metabolic flux analysis, should be carried out to identify bottlenecks in butanol assimilation to advance future host engineering. Our findings lay the groundwork for these studies by mapping the possible pathway intermediates and candidate genes responsible for each step of butanol assimilation (Fig. 4).

### Butanol affects the energetic state of the cell

**Figure 5:**
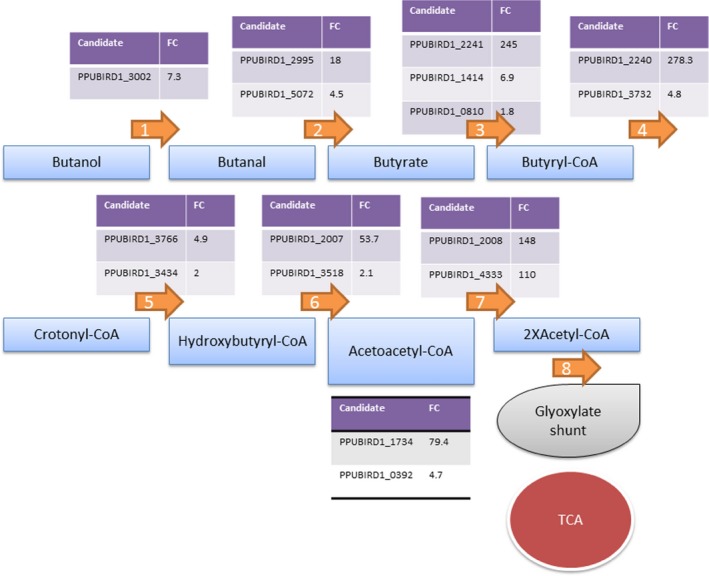
Butanol response model of the multifactorial strategies used to bypass butanol toxicity by *P*. *putida* BIRD‐1. The model shows different factors affected under butanol pressure as membrane, central metabolism and cofactor synthesis.

A set of genes involved in butanol tolerance and assimilation were identified by the construction of a mutant library and through selection of deficient mutants (Fig. 5). Many of the identified genes were involved in energy metabolism – with functions specifically related to the TCA cycle. This finding highlights the high energy levels required by cellular functions involved in the solvent stress response. For example, the resistance nodulation division (RND) efflux transporters TtgABC and MexEF, which, as previously discovered, serve as a major defense mechanism against solvents such as toluene (Ramos *et al*., 1998; Guazzaroni *et al*., 2005). We also found that the repressor of the TetR family (PPUBIRD1_2078) was found to be downregulated in transcriptomic and proteomic data. This repressor is involved in complex circuit regulation for various cellular functions, including multi‐drug efflux pumps systems (Ramos *et al*., 2005). We found that it was downregulated, which would be expected to induce efflux pump genes and concomitantly enhance butanol tolerance.

Genes capable of catalysing the conversion of ketoglutarate to succinyl‐CoA and NADH were also identified. These include LpdG, PPUBIRD1_1664 and SucA, which are key players in feeding electrons to cytochrome C (cellular redox status control). In this regard, our data also show that cytochrome C oxidase was upregulated in transcriptomic and proteomic analyses. Regarding transcriptomic analysis, we only found upregulation of CyoD after a butanol shock satisfying our fold‐change and *P*‐value criteria. However, in the proteomic analysis, several genes of the operon were found to be upregulated. We obtained a mutant in *aceF*, which encodes the E2 component of pyruvate dehydrogenase. In this mutant also acetyl‐CoA generation is altered and hence the energy generation, leading in turn to solvent sensitivity.

### Other relevant features

A gene strongly modulated by the presence of butanol was *rpoZ*. This gene encodes the omega subunit of RNA polymerase which is a complex that provides the cell with guanosine 3′,5′‐bispyrophosphate hydrolase activity and regulates a myriad of responses during conditions of stress (Fig. S2). Another important observation was that reduced production of proteins with enzymatic activity for (p)ppGpp biosynthesis conferred increased butanol tolerance. These results highlight an existing strategy for butanol production: bacterial strains with reduced (p)ppGpp accumulation combined with a functional butanol biosynthetic pathway have been developed and patented by DuPont (WO2009082681A1).

Cofactor biosynthesis – specifically thiamine biosynthesis – was also found to be altered in presence of butanol. Accordingly, we obtained two mutants in ApbE, a lipoprotein responsible of thiamine biosynthesis, and identified BioB as upregulated in our proteomic data for all the conditions. In support for a role for thiamine in butanol bioproduction, it has been shown to increase butanol titres in *S. cerevisiae* (US20120323047).

Regarding the *gluQ* mutant that we identified, there exists only one previous reference that links its upregulation to osmotic stress (Caballero *et al*., 2012). The authors of the study also showed that *glu*Q was downstream of *dks*A, a transcriptional regulator involved in osmotic stress response. It is worth to note that mutants in the biotin‐requiring 2‐oxoglutarate dehydrogenase complex were also butanol sensitive, linking the biotin deficiency in *P. putida* with energy generation.

As the pressure to develop quickly viable, renewable biofuel processes increases, a balance must be maintained between obtaining in‐depth biological knowledge and the application of that knowledge. Our data shed light on a great number of potential host engineering targets and provide a clearer understanding of butanol tolerance and assimilation. Recent advances in experimental and computational systems biology approaches could be used to complement these data to further refine our understanding of the cellular pathways governing butanol bio‐production.

## Experimental procedures

### Bacterial strains and culture conditions

The microorganisms used were *P. putida* BIRD‐1, a soil bacterium that is an efficient plant growth promoting rhizobacteria (Matilla *et al*., 2011), *P. putida* KT2440, a soil bacteria with generally regarded as safe (GRAS) status (Nakazawa, 2002), while *P. putida* DOT‐T1E is an aromatic hydrocarbon tolerant strain (Ramos *et al*., 1995). *Pseudomonas putida* was routinely grown in M9 minimal medium with glucose at 30°C and shaken at 200 r.p.m. When indicated, different industrial substrates were assayed as carbon sources using M9 minimal medium (Abril *et al*., 1989). These compounds were added according to the number of carbon per mol: succinate (0.665% v/v), glucose (0.5% v/v), lactate (1% w/v) and glycerol (1% w/v). Antibiotics were added, when necessary, to the culture medium to reach the following final concentrations (mg L^−1^): chloramphenicol (Cm), 30; kanamycin (Km), 25; rifampicin (Rif), 30.

Growth was monitored by measuring turbidity at 660 nm. To determine viable cells after a sudden butanol shock, *P. putida* was grown overnight in LB medium. The following day, cultures were diluted to reach a turbidity of 0.05 and allowed to grow until they reached about 0.8 (OD_660nm_). Subsequently, the cultures were split in two and 2% (v/v) of butanol was added to one of them, while the other was used as a control. The number of viable cells at different times after butanol addition was determined by drop plating at the proper dilutions. All experiments were performed in duplicate three times (Filloux, 2014).

### Mutagenesis

MiniTn*5*‐Km transposon mutagenesis was performed using triparental mating between the recipient (*P. putida* BIRD‐1), donor (*E. coli* CC118λ*pir*bearing pUT‐Km) and the helper *E. coli* HB101 with pRK600 (de Lorenzo and Timmis, 1994). After overnight incubation, equal volumes of the three strains were collected by centrifugation and suspended in fresh LB medium (500 μL). Spots containing equal concentrations of the three strains were placed on the surface of 0.45 μm filters on LB plates and incubated for 6 h at 30°C before being re‐suspended in minimal medium. To select transconjugants, the optimal dilution was plated on M9 minimal medium supplemented with Km and Rif and sodium benzoate 10 mM (as carbon source). The mutant clones selected (7860) were ordered in 384‐well plates by using a QPix2 robot (Genetix).

### Screening and identification of clones with specific phenotypes

For the screening, the mutant collection was transferred using QPix2 (Genetix) to plates containing the following media: LB; LB with butanol 0.7% (v/v); minimal medium M9 with glucose 0.5% (w/v); minimal medium M9 with glucose 0.5% (w/v) and butanol 0.7% (v/v); and minimal medium M9 with 0.5% (v/v) butanol as sole carbon source. To identify butanol sensitive mutants, LB and M9 glucose media were used in presence of the previously indicated butanol concentrations. Conversely, to identify mutants deficient in butanol assimilation, mutants that grew with glucose but failed to use butanol as the sole carbon source were selected.

To identify the points of mini‐transposon insertions (Caetano‐Anolles, 1993; O'Toole and Kolter, 1998) in BIRD‐1 mutants, we performed arbitrary PCR using *Taq* polymerase (Euroclone), using primer TNINT (5′‐AGGCGatttcagcgaagcac‐3′) (Sigma) (Ramos *et al*., 2007). The amplified DNA was submitted to Sanger sequencing in a 3130xl sequencer (Applied Biosystems). Sequences were analysed using the blastn algorithm (http://blast.ncbi.nlm.nih.gov/Blast.cgi).

### RNA isolation

To study the *P. putida* BIRD‐1 transcriptome under different conditions, we supplemented M9 minimal medium with glucose (0.5% w/v) (control), glucose (0.5% w/v) and butanol (0.3% v/v) or only butanol (0.3% v/v). A shock of butanol (0.5% v/v) was given for 1 h to cultures in the exponential growth phase (A_660nm_ = 0.8) while growing on glucose. At least two independent biological replicates were done. Cultures were harvested by adding and mixing 0.2 volumes of Stop Solution (95% ethanol, 5% phenol). Cells were pelleted by centrifugation (10 000 r.p.m. in a benchtop Eppendorf centrifuge). Total RNA was extracted with TRIzol (Invitrogen). Removal of DNA was carried out by DNase I treatment (Fermentas) in combination with the RNase inhibitor RiboLock (Fermentas). The integrity of total RNA and the presence of 5S ribosomal (r)RNA and DNA contamination were assessed with an Agilent 2100 Bioanalyzer (Agilent Technologies). Thereafter, the 23S, 16S and 5S rRNAs were removed by subtractive hybridization using the MICROBExpress kit (Ambion). Capture oligonucleotides were designed to be specifically complementary to the rRNAs in *Pseudomonas* (Gomez‐Lozano *et al*., 2014). Removal of rRNAs was confirmed with an Agilent 2100 Bioanalyzer (Agilent Technologies).

The sequencing libraries were prepared using the TruSeq kit (Illumina). First, the rRNA‐depleted RNA was fragmented using divalent cations under elevated temperature. The cleaved RNA fragments were copied into complementary (c)DNA using reverse transcriptase and random primers, followed by second‐strand cDNA synthesis using DNA polymerase I and RNase H. After this step, transcripts shorter than 100 nt were removed using Agencourt AMPure XP beads (Beckman Coulter Genomics). The remaining cDNA fragments were then subjected to an end‐repair process: the 3′‐addition of single ‘A’ bases and adapter ligation. This was followed by product purification and PCR amplification to generate the final cDNA library. The libraries were sequenced using the Illumina HiSeq2000 platform with a single‐end protocol and read lengths of 100 nucleotides.

### Rockhopper analysis

Considering all the samples and replicates, a total number of 34 267 239 reads were recorded to achieve an average sequence mapping for 91.5% of the cases. The average length of sequences was 100 bp. The reads were mapped onto the *P. putida* BIRD‐1 annotated reference genome (GenBank accession no. NC_017530) using rockhopper software (McClure *et al*., 2013) that is based on bowtie 2. For visualization, we used igv software (Robinson *et al*., 2011), which allowed us to study expression of RNAs and mRNAs within their genomic context.

Expression values reported by rockhopper for each transcript in each condition were normalized by the upper quartile of gene expression. A two‐sample Student's *t*‐test was performed on the average expression of the mRNAs to determine those with differential expression between the two conditions tested (*P*‐value < 0.02 and twofold change). To create a heat map, the Benjamini–Hochberg multiple testing correction was applied (Benjamini *et al*., 2001) when more than two samples were compared (*P*‐value < 0.05). Heat maps and hierarchical cluster analysis were created based on expression levels (*P*‐value < 0.05) using r.

### RNA sequencing data accession number

The sequence reads have been deposited in the GEO database under study accession no. GSE66235.

### Proteomics

To study the proteome of *P. putida* BIRD‐1, we used the same physiological conditions as for transcriptomics analysis, but three independent biological replicates were considered. Cells were collected by centrifugation at 10 000 × *g* for 2 min and washed with M9 medium without any carbon source and then pellets were stored at −80°C.

For the preparation of protein extracts, cell pellets were suspended in five volumes of sodium phosphate buffer 100 mM pH 8.2 with Complete Protease Inhibitor (1 tablet per 42 ml, Roche). Cells were lysed at 4°C by sonication applying a 40 J dose with amplitude of vibration of 30% and pulses of 10 s followed by resting intervals of 5 s using the UP50H Ultrasonic Processor (Hielscher Ultrasonics GmbH; max. output 45W) sonicator. Lysates were centrifuged for 20 min at 14 000 × *g* at 4°C to remove cellular debris. Protein content from the resulting soluble fractions was quantified by the Bradford‐based protein assay kit (BioRad). Lithium dodecyl sulphate‐β‐mercaptoethanol (LDS) protein gel sample buffer (Invitrogen) was added to the protein fractions at a ratio of 10 μL per 50 μg of protein. For the membrane protein‐specific fraction, the 12 pellets of cell debris were suspended in 1 ml of phosphate buffer. The samples were centrifuged for 30 min at 13 000 × *g*, and the pelleted material was washed twice with phosphate buffer to eliminate cytosolic contaminant proteins. The final pellets were suspended in 20 μL of LDS protein gel sample buffer. The soluble protein samples and the membrane protein‐specific fractions were then incubated at 99°C for 5 min prior to SDS‐PAGE.

### SDS‐PAGE and tandem mass spectrometry

Amounts of 50 μg of soluble protein and membrane protein fractions extracted from 100 mg cellular material (wet weight) were loaded on NuPAGE Novex 4–12% Bis‐Tris 1.5 mM, 10 wells gels (Invitrogen) for medium and short electrophoresis migrations respectively. The gels were run with MES buffer at 200 V and then stained with Coomasie Blue Safe stain. After overnight de‐staining, the whole protein content from each well was excised as seven polyacrylamide bands for soluble proteins and one band for the membrane proteins. These bands were de‐stained, and their protein contents were reduced and alkylated using iodoacetamide as previously described (Hartmann and Armengaud, 2014). The samples were proteolysed with sequencing‐grade Trypsin Gold and ProteaseMax surfactant (Promega). Digestion was stopped after 1 h at 50°C by adding 0.5% (v/v) trifluoroacetic acid to the samples. Tandem mass spectrometry analysis was performed on an LTQ Orbitrap XL (Thermo Fisher Scientific) coupled with an UltiMate 3000 LC system (Dionex), reverse‐phase Acclaim PepMap100 C18 μ‐precolumn (5 μm, 100 Å, 300 μm inner diameter × 5 mm, Dionex) and a nanoscale Acclaim PepMap100 C18 capillary column (3 μm, 100 Å, 75 μm i.d. × 15 cm, Dionex) as described previously (Clair *et al*., 2012). Sample loading volumes were 5 μL to prevent saturation. Polydimethylcyclosiloxane ions {monoprotonated [(CH_3_)_2_SiO)] 6 with m/z at 445.120024} from ambient air were used for internal recalibration in real time.

### MS/MS data processing

Peak lists were generated with the mascot daemon software (version 2.3.2; Matrix Science) using the extract_msn.exe data import filter (Thermo Fisher Scientific) from the xcalibur FT package (version 2.0.7; Thermo Fisher Scientific). Data import filter options were set to 400 (minimum mass), 5000 (maximum mass), 0 (grouping tolerance), 0 (intermediate scans) and 1000 (threshold) as described previously (Christie‐Oleza *et al*., 2012a). The mgf files from each sample were merged and MS/MS spectra were assigned using the mascot daemon 2.3.2 (Matrix Science) and the database containing the non‐redundant RefSeq protein entries for *P. putida* BIRD‐1 comprising 4960 protein sequences totalling 1 656 176 amino acids (National Center for Biotechnology Information download, 2014/01/07). The search was performed using the following criteria: tryptic peptides with a maximum of two miscleavages, mass tolerances of 5 ppm on the parent ion and 0.5 Da on the MS/MS, fixed modification for carbamidomethylated cysteine and variable modification for methionine oxidation. Mascot results were parsed using the irma 1.28.0 software (Dupierris *et al*., 2009). Peptides were identified with a *P*‐value threshold below 0.05. Proteins were considered validated when at least two distinct peptides were detected. The false discovery rate for protein identification was estimated with a reversed decoy database to be less than 1% using these parameters. Proteins were compared based on their spectral counts using the TFold Test using patternlab v2.0 (Carvalho *et al*., 2008; 2012) with a false discovery rate (Benjamini‐Hochberg q‐value) fixed at 0.05 and an F‐stringency set to 0.03. The NSAF was calculated by dividing the spectral count for each observed protein by its molecular weight expressed in kDa as previously described (Christie‐Oleza *et al*., 2012bb).

### Bioinformatics

Predictions for subcellular localization, clusters of orthologous groups (COG) number and COG functional category were obtained from the Pseudomonas Genome Database (http://www.pseudomonas.com/viewAllGenomes.do). Functional connections between proteins were analysed with the multiple sequences module from the STRING‐DB tools (http://string‐db.org/) after extracting their respective COG numbers. The highest confidence level (0.900) was applied for the network display (Franceschini *et al*., 2013).

### Data repository

The mass spectrometry proteomics data have been deposited to the ProteomeXchange Consortium (REFERENCE PMID:24727771) via the PRIDE partner repository with the data set identifier PXD002655 and 10.6019/PXD002655 (membrane proteins) and the data set identifier PXD002679 and 10.6019/PXD002679 (soluble proteins).

## Conflict of Interest

Authors declare no conflict of interest.

## Supporting information


**Fig. S1.** Cell death kinetics after a butanol shock of BIRD‐1, KT2440 and DOT‐T1E. Killing kinetics of *P. putida* strains upon exposure to different butanol concentrations. The strains were grown to reach the exponential phase (turbidity of 0.85 at 660 nm). At *t* = 0 the culture was divided into two aliquots, to which 1% or 2% (v/v) butanol was added. At the indicated times, the number of viable cells were estimated by plating dilutions on LB.
**Fig. S2.** ppGpp response model. ppGpp accumulation is mediated by the SpoT protein. In the genome, *spoT* is located downstream of *rpoZ*, which is the omega subunit of RNA polymerase.
**Table S1.** Doubling time of *P. putida* BIRD‐1, KT2440 and DOT‐T1E growing on different media. Doubling times (G) and lag phases (lag) are indicated.
**Table S2.** Mutant library characteristics and phenotypes. Mutants in a mutant library, insertion points of the sequences obtained and phenotype (A, assimilation, T, tolerance and A&T, assimilation and tolerance).
**Table S3.** Venn Diagram specification. Butanol as sole carbon source, Shock and glucose butanol grown cells. Each transcript found in common in the diagram is categorized.
**Table S4.** Transcriptomics results. Table obtained after comparison of the all the conditions versus the control (glucose grown cells).
**Table S5.** Peptides whole cell proteome detected by MS/MS List of redundant peptides obtained from whole cell proteome of the three biological replicates of the control (C), butanol grown cells (B), glucose plus butanol grown cells (GB) and cells after a butanol shock (S).
**Table S6.** Pattern Lab analysis of whole cell proteome. List of proteins from whole cells of *P. putida* BIRD‐1 validated with at least two different peptides.
**Table S7.** Peptides membrane proteome detected by MS/MS. List of redundant peptides obtained from membrane proteins of the three biological replicates of the control (C), butanol grown cells (B), glucose plus butanol grown cells (GB) and cells after a butanol shock (S).
**Table S8.** Pattern Lab analysis of membrane proteome. List of proteins from membrane of *P. putida* BIRD‐1 validated with at least two different peptides.

## References

[mbt212328-bib-0001] Abril, M.A. , Michan, C. , Timmis, K.N. , and Ramos, J.L. (1989) Regulator and enzyme specificities of the TOL plasmid‐encoded upper pathway for degradation of aromatic hydrocarbons and expansion of the substrate range of the pathway. J Bacteriol 171: 6782–6790. 2687253 10.1128/jb.171.12.6782-6790.1989 PMC210577

[mbt212328-bib-0002] Arp, D.J. (1999) Butane metabolism by butane‐grown *Pseudomonas butanovora* . Microbiology 145: 1173–1180. 10376833 10.1099/13500872-145-5-1173

[mbt212328-bib-0003] Atsumi, S. , Cann, A.F. , Connor, M.R. , Shen, C.R. , Smith, K.M. , Brynildsen, M.P. , *et al* (2008) Metabolic engineering of *Escherichia coli* for 1‐butanol production. Metab Eng 10: 305–311. 17942358 10.1016/j.ymben.2007.08.003

[mbt212328-bib-1001] Benjamini, Y. , and Yekutieli, D. (2001) The control of the false discovery rate in multiple testing under dependency. Ann Stat 29: 1165–1188.

[mbt212328-bib-0004] Berezina, O.V. , Zakharova, N.V. , Brandt, A. , Yarotsky, S.V. , Schwarz, W.H. , and Zverlov, V.V. (2010) Reconstructing the clostridial n‐butanol metabolic pathway in *Lactobacillus brevis* . Appl Microbiol Biotechnol 87: 635–646. 20195860 10.1007/s00253-010-2480-z

[mbt212328-bib-0005] Brynildsen, M.P. , and Liao, J.C. (2009) An integrated network approach identifies the isobutanol response network of *Escherichia coli* . Molecular System Biology 277: 1–13. 10.1038/msb.2009.34 PMC2710865 19536200

[mbt212328-bib-0006] Caballero, V.C. , Toledo, V.P. , Maturana, C. , Fisher, C.R. , Payne, S.M. , and Salazar, J.C. (2012) Expression of *Shigella flexneri* gluQ‐rs gene is linked to dksA and controlled by a transcriptional terminator. BMC Microbiol 12: 226. 23035718 10.1186/1471-2180-12-226 PMC3542578

[mbt212328-bib-0007] Caetano‐Anolles, G. (1993) Amplifying DNA with arbitrary oligonucleotide primers. PCR Methods Appl 3: 85–94. 8268791 10.1101/gr.3.2.85

[mbt212328-bib-0008] Carvalho, P. , Fischer, J. , Chen, E. , Yates, J. , and Barbosa, V. (2008) PatternLab for proteomics: a tool for differential shotgun proteomics. BMC Bioinformatics 9: 316. 18644148 10.1186/1471-2105-9-316 PMC2488363

[mbt212328-bib-0009] Carvalho, P.C. , Yates, J.R. , and Barbosa, V.C. (2012) Improving the TFold test for differential shotgun proteomics. Bioinformatics 28: 1652–1654. 22539673 10.1093/bioinformatics/bts247 PMC3371870

[mbt212328-bib-0010] Cascone, R. (2008) Biobutanol: A Replacement for Bioethanol? New York, NY, USA: American Institute of Chemical Engineers.

[mbt212328-bib-0011] Christie‐Oleza, J.A. , Fernandez, B. , Nogales, B. , Bosch, R. , and Armengaud, J. (2012a) Proteomic insights into the lifestyle of an environmentally relevant marine bacterium. ISME J 6: 124–135. 21776030 10.1038/ismej.2011.86 PMC3246242

[mbt212328-bib-0012] Christie‐Oleza, J.A. , Pina‐Villalonga, J.M. , Bosch, R. , Nogales, B. , and Armengaud, J. (2012b) Comparative proteogenomics of twelve *Roseobacter* exoproteomes reveals different adaptive strategies among these marine bacteria. Mol Cell Proteomics 11: 12–23. 10.1074/mcp.M111.013110 PMC3277765 22122883

[mbt212328-bib-0013] Clair, G. , Armengaud, J. , and Duport, C. (2012) Restricting fermentative potential by proteome remodeling: an adaptive strategy evidenced in *Bacillus cereus* . Mol Cell Proteomics 11. doi: 10.1074/mcp.M111.013102. 10.1074/mcp.M111.013102 PMC3433916 22232490

[mbt212328-bib-0014] Dominguez‐Cuevas, P. , Gonzalez‐Pastor, J.E. , Marques, S. , Ramos, J.L. , and de Lorenzo, V. (2006) Transcriptional tradeoff between metabolic and stress‐response programs in *Pseudomonas putida* KT2440 cells exposed to toluene. J Biol Chem 281: 11981–11991. 16495222 10.1074/jbc.M509848200

[mbt212328-bib-0015] Dupierris, V. , Masselon, C. , Court, M. , Kieffer‐Jaquinod, S. , and Bruley, C. (2009) A toolbox for validation of mass spectrometry peptides identification and generation of database: IRMa. Bioinformatics 25: 1980–1981. 19420053 10.1093/bioinformatics/btp301

[mbt212328-bib-0016] Dürre, P. (2011) Fermentative production of butanol: the academic perspective. Curr Opin Biotechnol 22: 331–336. 21565485 10.1016/j.copbio.2011.04.010

[mbt212328-bib-0017] Ezeji, T.C. , Qureshi, N. , and Blaschek, H.P. (2007) Bioproduction of butanol from biomass: from genes to bioreactors. Curr Opin Biotechnol 18: 220–227. 17462877 10.1016/j.copbio.2007.04.002

[mbt212328-bib-0018] Filloux, A.R.J.L. (2014) Pseudomonas: Methods and Protocols. New York, USA; Springer.

[mbt212328-bib-0019] Franceschini, A. , Szklarczyk, D. , Frankild, S. , Kuhn, M. , Simonovic, M. , Roth, A. , *et al* (2013) STRING v9.1: protein–protein interaction networks, with increased coverage and integration. Nucleic Acids Res 41: D808–D815. 23203871 10.1093/nar/gks1094 PMC3531103

[mbt212328-bib-0020] Gentry, D.R. , and Cashel, M. (1996) Mutational analysis of the *Escherichia coli* spoT gene identifies distinct but overlapping regions involved in ppGpp synthesis and degradation. Mol Microbiol 19: 1373–1384. 8730877 10.1111/j.1365-2958.1996.tb02480.x

[mbt212328-bib-0021] Gomez‐Lozano, M. , Marvig, R. , Tulstrup, M. , and Molin, S. (2014) Expression of antisense small RNAs in response to stress in *Pseudomonas aeruginosa* . BMC Genomics 15: 783. 25213728 10.1186/1471-2164-15-783 PMC4180829

[mbt212328-bib-0022] Green, E.M. (2011) Fermentative production of butanol – the industrial perspective. Curr Opin Biotechnol 22: 337–343. 21367598 10.1016/j.copbio.2011.02.004

[mbt212328-bib-0023] Guazzaroni, M.E. , Krell, T. , Felipe, A. , Ruiz, R. , Meng, C. , Zhang, X. , *et al* (2005) The multidrug efflux regulator TtgV recognizes a wide range of structurally different effectors in solution and complexed with target DNA: evidence from isothermal titration calorimetry. J Biol Chem 280: 20887–20893. 15767250 10.1074/jbc.M500783200

[mbt212328-bib-0024] Gulevich, A. , Skorokhodova, A. , Sukhozhenko, A. , Shakulov, R. , and Debabov, V. (2012) Metabolic engineering of *Escherichia coli* for 1‐butanol biosynthesis through the inverted aerobic fatty acid β‐oxidation pathway. Biotechnol Lett 34: 463–469. 22105550 10.1007/s10529-011-0797-z

[mbt212328-bib-0025] Hartmann, E.M. , and Armengaud, J. (2014) Shotgun proteomics suggests involvement of additional enzymes in dioxin degradation by *Sphingomonas wittichii* RW1. Environ Microbiol 16: 162–176. 24118890 10.1111/1462-2920.12264

[mbt212328-bib-0026] Lin, P.P. , Rabe, K.S. , Takasumi, J.L. , Kadisch, M. , Arnold, F.H. , and Liao, J.C. (2014) Isobutanol production at elevated temperatures in thermophilic *Geobacillus thermoglucosidasius* . Metab Eng 24: 1–8. 24721011 10.1016/j.ymben.2014.03.006

[mbt212328-bib-0027] Llamas, M.A. , Rodriguez‐Herva, J.J. , Hancock, R.E. , Bitter, W. , Tommassen, J. , and Ramos, J.L. (2003) Role of *Pseudomonas putida* tol‐oprL gene products in uptake of solutes through the cytoplasmic membrane. J Bacteriol 185: 4707–4716. 12896989 10.1128/JB.185.16.4707-4716.2003 PMC166457

[mbt212328-bib-0028] de Lorenzo, V. , and Timmis, K.N. (1994) Analysis and construction of stable phenotypes in gram‐negative bacteria with Tn*5*‐ and Tn*10*‐derived minitransposons. Methods Enzymol 235: 386–405. 8057911 10.1016/0076-6879(94)35157-0

[mbt212328-bib-0029] McClure, R. , Balasubramanian, D. , Sun, Y. , Bobrovskyy, M. , Sumby, P. , Genco, C.A. , *et al* (2013) Computational analysis of bacterial RNA‐Seq data. Nucleic Acids Res 41: e140. 23716638 10.1093/nar/gkt444 PMC3737546

[mbt212328-bib-0030] Madhusudhan, K.T. , Lorenz, D. , and Sokatch, J.R. (1993) The bkdR gene of *Pseudomonas putida* is required for expression of the bkd operon and encodes a protein related to Lrp of *Escherichia coli* . J Bacteriol 175: 3934–3940. 8320210 10.1128/jb.175.13.3934-3940.1993 PMC204820

[mbt212328-bib-0031] Martinez‐Granero, F. , Redondo‐Nieto, M. , Vesga, P. , Martin, M. , and Rivilla, R. (2014) AmrZ is a global transcriptional regulator implicated in iron uptake and environmental adaption in *P. fluorescens* F113. BMC Genomics 15: 237. 24670089 10.1186/1471-2164-15-237 PMC3986905

[mbt212328-bib-0032] Mathew, R. , Ramakanth, M. , and Chatterji, D. (2005) Deletion of the gene rpoZ, encoding the α subunit of RNA polymerase, in *Mycobacterium smegmatis* results in fragmentation of the ß’ subunit in the enzyme assembly. J Bacteriol 187: 6565–6570. 16159791 10.1128/JB.187.18.6565-6570.2005 PMC1236636

[mbt212328-bib-0033] Matilla, M.A. , Pizarro‐Tobias, P. , Roca, A. , Fernandez, M. , Duque, E. , Molina, L.Z. , *et al* (2011) Complete genome of the plant growth‐promoting rhizobacterium *Pseudomonas putida* BIRD‐1. J Bacteriol 193: 1290. 21183676 10.1128/JB.01281-10 PMC3067593

[mbt212328-bib-0034] Molina‐Santiago, C. , Daddaoua, A. , Fillet, S. , Duque, E. , and Ramos, J.‐L. (2014) Interspecies signalling: pseudomonas putida efflux pump TtgGHI is activated by indole to increase antibiotic resistance. Environ Microbiol 16: 1267–1281. 24373097 10.1111/1462-2920.12368

[mbt212328-bib-0035] Mukherjee, K. , Nagai, H. , Shimamoto, N. , and Chatterji, D. (1999) GroEL is involved in activation of *Escherichia coli* RNA polymerase devoid of the ω subunit in vivo. Eur J Biochem 266: 228–235. 10542069 10.1046/j.1432-1327.1999.00848.x

[mbt212328-bib-0036] Nakazawa, T. (2002) Travels of a *Pseudomonas*, from Japan around the world. Environ Microbiol 4: 782–786. 12534461 10.1046/j.1462-2920.2002.00310.x

[mbt212328-bib-0037] Nielsen, D.R. , Leonard, E. , Yoon, S.‐H. , Tseng, H.‐C. , Yuan, C. , and Prather, K.L.J. (2009) Engineering alternative butanol production platforms in heterologous bacteria. Metab Eng 11: 262–273. 19464384 10.1016/j.ymben.2009.05.003

[mbt212328-bib-0038] O'Toole, G.A. , and Kolter, R. (1998) Initiation of biofilm formation in *Pseudomonas fluorescens* WCS365 proceeds via multiple, convergent signalling pathways: a genetic analysis. Mol Microbiol 28: 449–461. 9632250 10.1046/j.1365-2958.1998.00797.x

[mbt212328-bib-0039] Papoutsakis, E.T. , and Alsaker, K.V. (2012) Towards a synthetic biology of the stress‐response and the tolerance phenotype: systems understanding and engineering of the *Clostridium acetobutylicum* stress‐response and tolerance to toxic metabolites In Systems Metabolic Engineering. Netherlands: Springer, pp. 193–219.

[mbt212328-bib-0040] Ramos, J.‐L. , Filloux, A. , Duque, E. , Molina‐Henares, A. , Torre, J.S. , Molina‐Henares, M.A. , *et al* (2007) Towards a genome‐wide mutant library of *Pseudomonas putida* strain KT2440 In Pseudomonas. Netherlands: Springer, 227–251.

[mbt212328-bib-0041] Ramos, J.L. , Duque, E. , Huertas, M.J. , and Haidour, A. (1995) Isolation and expansion of the catabolic potential of a *Pseudomonas putida* strain able to grow in the presence of high concentrations of aromatic hydrocarbons. J Bacteriol 177: 3911–3916. 7608060 10.1128/jb.177.14.3911-3916.1995 PMC177117

[mbt212328-bib-0042] Ramos, J.L. , Duque, E. , Rodriguez‐Herva, J.J. , Godoy, P. , Haidour, A. , Reyes, F. , and Fernandez‐Barrero, A. (1997) Mechanisms for solvent tolerance in bacteria. J Biol Chem 272: 3887–3890. 9020089 10.1074/jbc.272.7.3887

[mbt212328-bib-0043] Ramos, J.L. , Duque, E. , Godoy, P. , and Segura, A. (1998) Efflux pumps involved in toluene tolerance in *Pseudomonas putida* DOT‐T1E. J Bacteriol 180: 3323–3329. 9642183 10.1128/jb.180.13.3323-3329.1998 PMC107285

[mbt212328-bib-0044] Ramos, J.L. , Duque, E. , Gallegos, M.T. , Godoy, P. , Ramos‐Gonzalez, M.I. , Rojas, A. , *et al* (2002) Mechanisms of solvent tolerance in gram‐negative bacteria. Annu Rev Microbiol 56: 743–768. 12142492 10.1146/annurev.micro.56.012302.161038

[mbt212328-bib-0045] Ramos, J.L. , Martinez‐Bueno, M. , Molina‐Henares, A.J. , Teran, W. , Watanabe, K. , Zhang, X. , *et al* (2005) The TetR family of transcriptional repressors. Microbiol Mol Biol Rev 69: 326–356. 15944459 10.1128/MMBR.69.2.326-356.2005 PMC1197418

[mbt212328-bib-0046] Ramos, J.L. , Sol Cuenca, M. , Molina‐Santiago, C. , Segura, A. , Duque, E. , Gomez‐Garcia, M.R. , *et al* (2015) Mechanisms of solvent resistance mediated by interplay of cellular factors in *Pseudomonas putida* . FEMS Microbiol Rev. doi: 10.1007/s00253‐015‐6963‐9. 10.1093/femsre/fuv006 25934123

[mbt212328-bib-0047] Reyes, L.H. , Almario, M.P. , and Kao, K.C. (2011) Genomic library screens for genes involved in n‐butanol tolerance in *Escherichia coli* . PLoS ONE 6: e17678. 21408113 10.1371/journal.pone.0017678 PMC3050900

[mbt212328-bib-0048] Robinson, J.T. , Thorvaldsdottir, H. , Winckler, W. , Guttman, M. , Lander, E.S. , Getz, G. , and Mesirov, J.P. (2011) Integrative genomics viewer. Nat Biotechnol 29: 24–26. 21221095 10.1038/nbt.1754 PMC3346182

[mbt212328-bib-0049] Rutherford, B.J. , Dahl, R.H. , Price, R.E. , Szmidt, H.L. , Benke, P.I. , Mukhopadhyay, A. , and Keasling, J.D. (2010) Functional genomic study of exogenous n‐butanol stress in *Escherichia coli* . Appl Environ Microbiol 76: 1935–1945. 20118358 10.1128/AEM.02323-09 PMC2838030

[mbt212328-bib-0050] Schiel‐Bengelsdorf, B. , Montoya, J. , Linder, S. , and Dürre, P. (2013) Butanol fermentation. Environ Technol 34: 1691–1710. 24350428 10.1080/09593330.2013.827746

[mbt212328-bib-0051] Steen, E. , Chan, R. , Prasad, N. , Myers, S. , Petzold, C. , Redding, A. , *et al* (2008) Metabolic engineering of *Saccharomyces cerevisiae* for the production of n‐butanol. Microb Cell Fact 7: 1–8. 19055772 10.1186/1475-2859-7-36 PMC2621116

[mbt212328-bib-0052] Udaondo, Z. , Duque, E. , Fernández, M. , Molina, L. , Torre, J.D.L. , Bernal, P. , *et al* (2012) Analysis of solvent tolerance in *Pseudomonas putida* DOT‐T1E based on its genome sequence and a collection of mutants. FEBS Lett 586: 2932–2938. 22819823 10.1016/j.febslet.2012.07.031

