# Supplementary material for: Understanding butanol tolerance and assimilation in P seudomonas putida BIRD‐1: an integrated omics approach
Source: Microb Biotechnol. 2016 Jan 6;9(1):100–15. doi: 10.1111/1751-7915.12328 (PMC4720416; doi:10.1111/1751-7915.12328)
Supplement: Supplementary file 1 — Fig. S1. Cell death kinetics after a butanol shock of BIRD‐1, KT2440 and DOT‐T1E. Killing kinetics of P. putida strains upon exposure to different butanol concentrations. The strains were grown to reach the exponential phase (turbidity of 0.85 at 660 nm). At t = 0 the culture was divided into two aliquots, to which 1% or 2% (v/v) butanol was added. At the indicated times, the number of viable cells were estimated by plating dilutions on LB. Fig. S2. ppGpp response model. ppGpp accumulation is mediated by the SpoT protein. In the genome, spoT is located downstream of rpoZ, which is the omega subunit of RNA polymerase. Table S1. Doubling time of P. putida BIRD‐1, KT2440 and DOT‐T1E growing on different media. Doubling times (G) and lag phases (lag) are indicated. Table S2. Mutant library characteristics and phenotypes. Mutants in a mutant library, insertion points of the sequences obtained and phenotype (A, assimilation, T, tolerance and A&T, assimilation and tolerance). Table S3. Venn Diagram specification. Butanol as sole carbon source, Shock and glucose butanol grown cells. Each transcript found in common in the diagram is categorized. Table S4. Transcriptomics results. Table obtained after comparison of the all the conditions versus the control (glucose grown cells). Table S5. Peptides whole cell proteome detected by MS/MS List of redundant peptides obtained from whole cell proteome of the three biological replicates of the control (C), butanol grown cells (B), glucose plus butanol grown cells (GB) and cells after a butanol shock (S). Table S6. Pattern Lab analysis of whole cell proteome. List of proteins from whole cells of P. putida BIRD‐1 validated with at least two different peptides. Table S7. Peptides membrane proteome detected by MS/MS. List of redundant peptides obtained from membrane proteins of the three biological replicates of the control (C), butanol grown cells (B), glucose plus butanol grown cells (GB) and cells after a butanol shock (S). Table S8. [file MBT2-9-100-s001.zip › Table S3.docx]

**Table S3. Venn Diagram**.

| S DOWN. Genes downregulated after a butanol shock | | | |  |
| --- | --- | --- | --- | --- |
| PPUBIRD1_2933 | | hypothetical protein | |  |
| PPUBIRD1_0465 | | Histidine triad (HIT) protein | |  |
| PPUBIRD1_0439 | | KsgA | |  |
| PPUBIRD1_0591 | | Ethanolamine ammonia-lyase light chain | |  |
| PPUBIRD1_1314 | | hypothetical protein | |  |
| PPUBIRD1_4379 | | protein IlvH | |  |
| PPUBIRD1_4701 | | hypothetical protein | |  |
| PPUBIRD1_2019 | | hypothetical protein | |  |
| PPUBIRD1_0679 | | hypothetical protein | |  |
| PPUBIRD1_5062 | | Cro/CI family transcriptional regulator | |  |
| PPUBIRD1_0355 | | SoxD | |  |
| PPUBIRD1_3202 | | hypothetical protein | |  |
| PPUBIRD1_0899 | | hypothetical protein | |  |
| PPUBIRD1_3298 | | hypothetical protein | |  |
| PPUBIRD1_0629 | | hypothetical protein | |  |
| PPUBIRD1_0389 | | DNA polymerase III subunit epsilon | |  |
| PPUBIRD1_4161 | | hypothetical protein | |  |
| PPUBIRD1_2709 | | Glutaredoxin | |  |
| PPUBIRD1_0506 | | protein RplF | |  |
| PPUBIRD1_4265 | | Carboxylesterase | |  |
| PPUBIRD1_1732 | | hypothetical protein | |  |
| PPUBIRD1_4604 | | Fis | |  |
| PPUBIRD1_1180 | | Membrane protein-like protein | |  |
| PPUBIRD1_4688 | | hypothetical protein | |  |
| PPUBIRD1_1261 | | OprL | |  |
| PPUBIRD1_0606 | | ATP-NAD/AcoX kinase | |  |
| PPUBIRD1_2823 | | hypothetical protein | |  |
| PPUBIRD1_0128 | | CynT | |  |
| PPUBIRD1_3620 | | ATPase | |  |
| PPUBIRD1_3934 | | MarR family transcriptional regulator | |  |
| PPUBIRD1_4719 | | O-antigen polymerase | |  |
| PPUBIRD1_3031 | | Helix-turn-helix domain-containing protein | |  |
| PPUBIRD1_4125 | | LepA protein | |  |
| PPUBIRD1_4551 | | hypothetical protein | |  |
| PPUBIRD1_0453 | | hypothetical protein | |  |
| PPUBIRD1_3739 | | hypothetical protein | |  |
| PPUBIRD1_2929 | | UspA domain-containing protein | |  |
| PPUBIRD1_1605 | | CcoO_2 | |  |
| PPUBIRD1_1165 | | hypothetical protein | |  |
| PPUBIRD1_0655 | | protein LspA | |  |
| PPUBIRD1_3204 | | Integrase family protein | |  |
| PPUBIRD1_4281 | | AlgI protein | |  |
| PPUBIRD1_4350 | | protein TrmA | |  |
| PPUBIRD1_5078 | | RadC | |  |
| PPUBIRD1_0988 | | hypothetical protein | |  |
| PPUBIRD1_2495 | | PaaH | |  |
| PPUBIRD1_4622 | | hypothetical protein | |  |
| PPUBIRD1_1219 | | hypothetical protein | |  |
| PPUBIRD1_2030 | | Enoyl-CoA hydratase/isomerase | |  |
| PPUBIRD1_3438 | | FadD protein | |  |
| PPUBIRD1_3550 | | Deoxyguanosinetriphosphate triphosphohydrolase-like protein | |  |
| PPUBIRD1_0319 | | protein HisH | |  |
| PPUBIRD1_2602 | | hypothetical protein | |  |
| PPUBIRD1_1259 | | Protein TolA | |  |
| PPUBIRD1_2053 | | CatA | |  |
| PPUBIRD1_4096 | | protein RimM | |  |
| PPUBIRD1_1799 | | HflD-like high frequency lysogenization protein | |  |
| PPUBIRD1_2412 | | Major facilitator family transporter | |  |
| PPUBIRD1_0473 | | TyrS | |  |
| PPUBIRD1_2585 | | Periplasmic polyamine-binding protein. putative | |  |
| PPUBIRD1_3076 | | Major facilitator family transporter | |  |
| PPUBIRD1_3046 | | Response regulator receiver/ANTAR domain-containing protein | |  |
| PPUBIRD1_1214 | | DctP | |  |
| PPUBIRD1_2484 | | Universal stress protein | |  |
| PPUBIRD1_3013 | | hypothetical protein | |  |
| PPUBIRD1_2402 | | Ribokinase-like domain-containing protein | |  |
| PPUBIRD1_0917 | | Anti-FecI sigma factor. FecR | |  |
| PPUBIRD1_1022 | | GntR family transcriptional regulator | |  |
| PPUBIRD1_1892 | | TetR family transcriptional regulator | |  |
| PPUBIRD1_3470 | | Cro/CI family transcriptional regulator | |  |
| PPUBIRD1_2334 | | Acyl-CoA synthetase | |  |
| PPUBIRD1_1545 | | hypothetical protein | |  |
| PPUBIRD1_3560 | | Nitrite transporter | |  |
| PPUBIRD1_4992 | | UbiF | |  |
| PPUBIRD1_4980 | | ArgA | |  |
| PPUBIRD1_3635 | | hypothetical protein | |  |
| PPUBIRD1_1488 | | Two component. sigma54 specific. Fis family transcriptional regulator | |  |
| PPUBIRD1_1777 | | Gnd | |  |
| PPUBIRD1_2184 | | Qor | |  |
| PPUBIRD1_4923 | | hypothetical protein | |  |
| PPUBIRD1_1070 | | aldose 1-epimerase | |  |
| PPUBIRD1_4202 | | GroES protein | |  |
| PPUBIRD1_0952 | | ColR | |  |
| PPUBIRD1_4846 | | Carboxyl-terminal protease | |  |
| PPUBIRD1_4565 | | ThiD | |  |
| PPUBIRD1_3558 | | Nitrate-binding protein NasS. putative | |  |
| PPUBIRD1_0517 | | protein RplQ | |  |
| PPUBIRD1_2153 | | ABC-type nitrate/sulfonate/bicarbonate transport systems periplasmic components-like protein | |  |
| PPUBIRD1_0912 | | TonB-dependent siderophore receptor | |  |
| PPUBIRD1_2135 | | GAF sensor hybrid histidine kinase | |  |
| PPUBIRD1_4445 | | LysR family transcriptional regulator | |  |
| PPUBIRD1_4757 | | hypothetical protein | |  |
| PPUBIRD1_4703 | | hypothetical protein | |  |
| PPUBIRD1_0429 | | Glycerol-3-phosphate acyltransferase | |  |
| PPUBIRD1_1155 | | ATP-dependent DNA ligase | |  |
| PPUBIRD1_3425 | | Putative monovalent cation/H+ antiporter subunit C | |  |
| PPUBIRD1_2407 | | Surface antigen (D15) | |  |
| PPUBIRD1_4974 | | PotG | |  |
| PPUBIRD1_2126 | | Phage integrase family protein | |  |
| PPUBIRD1_3156 | | hypothetical protein | |  |
| PPUBIRD1_2371 | | hypothetical protein | |  |
| PPUBIRD1_3256 | | Alcohol dehydrogenase | |  |
| PPUBIRD1_0415 | | PqqB | |  |
| PPUBIRD1_1038 | | GcvP | |  |
| PPUBIRD1_4339 | | uracil-xanthine permease | |  |
| PPUBIRD1_4222 | | protein FtsA | |  |
| PPUBIRD1_0234 | | hypothetical protein | |  |
| PPUBIRD1_4863 | | HemE protein | |  |
| PPUBIRD1_0948 | | Hydro-lyase. Fe-S type. tartrate/fumarate subfamily. alpha subunit | |  |
| PPUBIRD1_0284 | | protein FdhD | |  |
| PPUBIRD1_4907 | | Alpha/beta fold family hydrolase | |  |
| PPUBIRD1_4596 | | PAP2 family protein/DedA family protein | |  |
| PPUBIRD1_2294 | | Sigma54 specific transcriptional regulator. Fis family | |  |
| PPUBIRD1_2165 | | Gluconate 2-dehydrogenase acceptor subunit | |  |
| PPUBIRD1_3922 | | Integral membrane sensor hybrid histidine kinase | |  |
| PPUBIRD1_4952 | | Lysophospholipase-like protein | |  |
| PPUBIRD1_3461 | | hypothetical protein | |  |
| S UP. Genes upregulated after a butanol shock | | | |  |
| PPUBIRD1_2250 | GntR family transcriptional regulator | | |  |
| PPUBIRD1_2998 | Beta-lactamase domain protein | | |  |
| PPUBIRD1_1788 | lipocalin family protein | | |  |
| PPUBIRD1_0256 | TauD | | |  |
| PPUBIRD1_0302 | hypothetical protein | | |  |
| PPUBIRD1_1733 | hypothetical protein | | |  |
| PPUBIRD1_1505 | protein FliR | | |  |
| PPUBIRD1_2435 | hypothetical protein | | |  |
| PPUBIRD1_3923 | hypothetical protein | | |  |
| PPUBIRD1_4811 | Polar amino acid ABC transporter. inner membrane subunit | | |  |
| PPUBIRD1_3394 | sugar ABC transporter ATP-binding protein | | |  |
| PPUBIRD1_0859 | CyoD protein | | |  |
| B S DOWN. Common genes downregulated after a butanol shock and on cells growing on butanol as carbon source | | | |  |
| PPUBIRD1_1071 | DNA-binding transcriptional regulator HexR | | |  |
| PPUBIRD1_3437 | FadB2 | | |  |
| PPUBIRD1_0693 | ISPsy5. Orf1 | | |  |
| PPUBIRD1_2761 | hypothetical protein | | |  |
| PPUBIRD1_2685 | AroE_2 | | |  |
| PPUBIRD1_4233 | cell division protein FtsL | | |  |
| PPUBIRD1_5087 | hypothetical protein | | |  |
| PPUBIRD1_3520 | Universal stress protein | | |  |
| PPUBIRD1_1548 | mechanosensitive ion channel protein MscS | | |  |
| PPUBIRD1_3766 | Enoyl-CoA hydratase | | |  |
| PPUBIRD1_0832 | hypothetical protein | | |  |
| PPUBIRD1_4068 | Putative CheW protein | | |  |
| PPUBIRD1_3791 | glutathione S-transferase | | |  |
| PPUBIRD1_3798 | hypothetical protein | | |  |
| PPUBIRD1_2404 | gluconate 2-dehydrogenase | | |  |
| PPUBIRD1_3980 | hypothetical protein | | |  |
| PPUBIRD1_1388 | hypothetical protein | | |  |
| PPUBIRD1_3977 | Protein sprT | | |  |
| B S UP. Genes upregulated on cells after a butanol shock and on cells grown in butanol as carbon source | | | |  |
| PPUBIRD1_1827 | short-chain dehydrogenase | | |  |
| PPUBIRD1_2678 | hypothetical protein | | |  |
| B UP. Genes upregulated on cells grown in butanol as carbon source | | | | |
| PPUBIRD1_3684 | LysR family transcriptional regulator | |  |  |
| PPUBIRD1_1850 | Extracellular solute-binding protein | |  |  |
| PPUBIRD1_4272 | hypothetical protein | |  |  |
| PPUBIRD1_3556 | RlmL | |  |  |
| PPUBIRD1_4171 | Oxaloacetate decarboxylase | |  |  |
| PPUBIRD1_2631 | Major facilitator transporter | |  |  |
| PPUBIRD1_3959 | hypothetical protein | |  |  |
| PPUBIRD1_2063 | AraC family transcriptional regulator | |  |  |
| PPUBIRD1_1963 | binding-protein-dependent transport system inner membrane protein | |  |  |
| PPUBIRD1_3167 | Outer membrane porin | |  |  |
| PPUBIRD1_3580 | Ferric-pseudobactin M114 receptor pbuA | |  |  |
| PPUBIRD1_1681 | TonB-dependent receptor. plug | |  |  |
| PPUBIRD1_2140 | Aldehyde dehydrogenase | |  |  |
| PPUBIRD1_3877 | Beta (1-6) glucans synthase. putative | |  |  |
| PPUBIRD1_3463 | TRNA--hydroxylase | |  |  |
| PPUBIRD1_2673 | Alcohol dehydrogenase | |  |  |
| PPUBIRD1_3305 | hypothetical protein | |  |  |
| PPUBIRD1_4795 | protein PhaF | |  |  |
| PPUBIRD1_2186 | hypothetical protein | |  |  |
| PPUBIRD1_0964 | hypothetical protein | |  |  |
| PPUBIRD1_1541 | Hydantoin racemase. putative | |  |  |
| PPUBIRD1_0759 | Secretion protein HlyD family protein | |  |  |
| PPUBIRD1_3897 | Alcohol dehydrogenase. zinc-containing | |  |  |
| PPUBIRD1_1955 | hypothetical protein | |  |  |
| PPUBIRD1_3007 | YVTN family beta-propeller repeat-containing protein | |  |  |
| PPUBIRD1_2362 | MexF | |  |  |
| PPUBIRD1_2317 | Type II secretion system protein G | |  |  |
| PPUBIRD1_2286 | hypothetical protein | |  |  |
| PPUBIRD1_3375 | methyl-accepting chemotaxis sensory transducer | |  |  |
| PPUBIRD1_2749 | hypothetical protein | |  |  |
| PPUBIRD1_1521 | hypothetical protein | |  |  |
| PPUBIRD1_3075 | Fumarate reductase/succinate dehydrogenase flavoprotein domain protein | |  |  |
| PPUBIRD1_3883 | protein MinC | |  |  |
| PPUBIRD1_3903 | Peptidylprolyl isomerase FKBP-type | |  |  |
| PPUBIRD1_1985 | L-ornithine N5-oxygenase | |  |  |
| PPUBIRD1_1803 | isocitrate dehydrogenase | |  |  |
| PPUBIRD1_1808 | Putative arginyl-tRNA--protein transferase | |  |  |
| PPUBIRD1_3864 | Acetyltransferase | |  |  |
| PPUBIRD1_2043 | Periplasmic polyamine-binding protein. putative | |  |  |
| PPUBIRD1_3532 | Putative lipoprotein | |  |  |
| PPUBIRD1_3376 | hypothetical protein | |  |  |
| PPUBIRD1_2470 | protein MalK | |  |  |
| PPUBIRD1_3101 | hypothetical protein | |  |  |
| PPUBIRD1_2501 | PhaK | |  |  |
| PPUBIRD1_3261 | Anti-FecI sigma factor. FecR | |  |  |
| PPUBIRD1_2292 | hypothetical protein | |  |  |
| PPUBIRD1_2849 | Cytochrome B561 | |  |  |
| PPUBIRD1_2878 | hypothetical protein | |  |  |
| PPUBIRD1_2372 | GntP protein | |  |  |
| PPUBIRD1_2589 | LysR family transcriptional regulator | |  |  |
| PPUBIRD1_0531 | Formate dehydrogenase accessory protein FdhE | |  |  |
| PPUBIRD1_2177 | TonB-dependent siderophore receptor | |  |  |
| PPUBIRD1_1526 | protein CcmC | |  |  |
| PPUBIRD1_2211 | signaling protein | |  |  |
| PPUBIRD1_4941 | RpiA | |  |  |
| PPUBIRD1_3374 | TatD-related deoxyribonuclease | |  |  |
| PPUBIRD1_2223 | Acetylornithine deacetylase | |  |  |
| PPUBIRD1_3396 | Diguanylate cyclase/phosphodiesterase with PAS/PAC and GAF sensor(s) | |  |  |
| PPUBIRD1_2931 | Acetyltransferase | |  |  |
| PPUBIRD1_2180 | hypothetical protein | |  |  |
| PPUBIRD1_3011 | Two component LuxR family transcriptional regulator | |  |  |
| PPUBIRD1_3002 | QedH | |  |  |
| PPUBIRD1_2108 | Transcriptional regulator MvaT. P16 subunit. putative | |  |  |
| PPUBIRD1_4325 | MerR family transcriptional regulator | |  |  |
| PPUBIRD1_2619 | LexA repressor | |  |  |
| PPUBIRD1_2487 | PhaM | |  |  |
| PPUBIRD1_3003 | Pentapeptide repeat-containing protein | |  |  |
| PPUBIRD1_2374 | LacI family transcriptional regulator | |  |  |
| PPUBIRD1_1600 | CcoO | |  |  |
| PPUBIRD1_4681 | hypothetical protein | |  |  |
| PPUBIRD1_3045 | AmiS/UreI transporter | |  |  |
| PPUBIRD1_0117 | OsmC family protein | |  |  |
| PPUBIRD1_2235 | binding-protein-dependent transport system inner membrane protein | |  |  |
| PPUBIRD1_2350 | hypothetical protein | |  |  |
| PPUBIRD1_2615 | Aldo/keto reductase | |  |  |
| PPUBIRD1_2332 | hypothetical protein | |  |  |
| PPUBIRD1_1001 | PtsO | |  |  |
| PPUBIRD1_3004 | Two component LuxR family transcriptional regulator | |  |  |
| PPUBIRD1_2179 | hypothetical protein | |  |  |
| PPUBIRD1_2647 | BdhA | |  |  |
| PPUBIRD1_1728 | NADH dehydrogenase subunit E | |  |  |
| PPUBIRD1_3341 | hypothetical protein | |  |  |
| PPUBIRD1_t0026 | - | |  |  |
| PPUBIRD1_3000 | Extracellular solute-binding protein | |  |  |
| PPUBIRD1_2952 | hemerythrin HHE cation binding domain-containing protein | |  |  |
| PPUBIRD1_1170 | hypothetical protein | |  |  |
| PPUBIRD1_2478 | Lipoprotein OprI. putative | |  |  |
| PPUBIRD1_2386 | hypothetical protein | |  |  |
| B GB UP. Common genes upregulated on cells grown on butanol as carbón source and cells grown in glucose and butanol | | |  |  |
| PPUBIRD1_2640 | Phospho-2-dehydro-3-deoxyheptonate aldolase | |  |  |
| PPUBIRD1_1878 | hypothetical protein | |  |  |
| PPUBIRD1_2189 | GntR family transcriptional regulator | |  |  |
| PPUBIRD1_1326 | AAA ATPase | |  |  |
| PPUBIRD1_1842 | PcaI | |  |  |
| PPUBIRD1_0399 | protein BioB | |  |  |
| PPUBIRD1_4947 | hypothetical protein | |  |  |
| PPUBIRD1_3216 | hypothetical protein | |  |  |
| B DOWN. Genes downregulated in cells grown in butanol as carbon source | | | | |
| PPUBIRD1_1482 | hypothetical protein | |  |  |
| PPUBIRD1_2902 | LysR family transcriptional regulator | |  |  |
| PPUBIRD1_1062 | GltR_2 | |  |  |
| PPUBIRD1_4920 | hypothetical protein | |  |  |
| PPUBIRD1_4011 | protein LpxB | |  |  |
| PPUBIRD1_1330 | hypothetical protein | |  |  |
| PPUBIRD1_4502 | Putative type IV secretion system protein IcmC/DotIE | |  |  |
| PPUBIRD1_1824 | hypothetical protein | |  |  |
| PPUBIRD1_1286 | Amino acid transporter LysE | |  |  |
| PPUBIRD1_1583 | Major facilitator family transporter | |  |  |
| PPUBIRD1_1422 | AruF | |  |  |
| PPUBIRD1_1221 | hypothetical protein | |  |  |
| PPUBIRD1_3810 | protein KdsA | |  |  |
| PPUBIRD1_1942 | hypothetical protein | |  |  |
| PPUBIRD1_3835 | Glycosyltransferases involved in cell wall biogenesis | |  |  |
| PPUBIRD1_1131 | Glutaredoxin-like protein | |  |  |
| PPUBIRD1_4505 | Putative type IV secretion system protein IcmK/DotH | |  |  |
| PPUBIRD1_4548 | ATP-dependent helicase HrpB | |  |  |
| PPUBIRD1_5086 | hypothetical protein | |  |  |
| PPUBIRD1_0326 | Sda | |  |  |
| PPUBIRD1_3386 | hypothetical protein | |  |  |
| PPUBIRD1_0581 | hypothetical protein | |  |  |
| PPUBIRD1_4547 | hypothetical protein | |  |  |
| PPUBIRD1_0051 | Histidine kinase | |  |  |
| PPUBIRD1_0311 | GabP | |  |  |
| PPUBIRD1_0340 | Oxidoreductase. FMN-binding protein | |  |  |
| PPUBIRD1_0909 | Putative aminotransferase | |  |  |
| PPUBIRD1_2795 | hypothetical protein | |  |  |
| PPUBIRD1_0539 | hypothetical protein | |  |  |
| PPUBIRD1_4516 | Acyl-CoA thioesterase II | |  |  |
| PPUBIRD1_0041 | LysR family transcriptional regulator | |  |  |
| PPUBIRD1_3806 | Polysaccharide export protein | |  |  |
| PPUBIRD1_4916 | Putative signal transduction protein | |  |  |
| PPUBIRD1_3497 | Heavy metal sensor signal transduction histidine kinase | |  |  |
| PPUBIRD1_0758 | NodT family RND efflux system outer membrane lipoprotein | |  |  |
| PPUBIRD1_1110 | glutamate synthase (NADPH) | |  |  |
| PPUBIRD1_3718 | hypothetical protein | |  |  |
| PPUBIRD1_2794 | hypothetical protein | |  |  |
| PPUBIRD1_1246 | Cold-shock DNA-binding domain-containing protein | |  |  |
| PPUBIRD1_1917 | Lambda family phage tail tape measure protein | |  |  |
| PPUBIRD1_3395 | GAF modulated Fis family sigma-54 specific transcriptional regulator | |  |  |
| PPUBIRD1_3667 | hypothetical protein | |  |  |
| PPUBIRD1_1993 | hypothetical protein | |  |  |
| PPUBIRD1_0944 | Intracellular protease. PfpI family | |  |  |
| PPUBIRD1_4151 | PhaG | |  |  |
| PPUBIRD1_1150 | Dcd | |  |  |
| PPUBIRD1_4315 | Fumarylacetoacetase | |  |  |
| PPUBIRD1_0447 | PAS/PAC sensor signal transduction histidine kinase | |  |  |
| PPUBIRD1_0240 | Fatty acid desaturase | |  |  |
| PPUBIRD1_1179 | FAD dependent oxidoreductase | |  |  |
| GB UP. Genes downregulated in cells grown in glucose and butanol | | | | |
| PPUBIRD1_4467 | hypothetical protein | |  |  |
| PPUBIRD1_3471 | Putative aminotransferase | |  |  |
| PPUBIRD1_3331 | Multi-sensor signal transduction histidine kinase | |  |  |
| PPUBIRD1_2231 | hypothetical protein | |  |  |
| PPUBIRD1_2279 | 5-oxoprolinase | |  |  |
| PPUBIRD1_1958 | Cytochrome c. class I | |  |  |
| PPUBIRD1_3822 | hypothetical protein | |  |  |
| PPUBIRD1_2586 | Oxidoreductase. putative | |  |  |
| PPUBIRD1_3028 | LysR family transcriptional regulator | |  |  |
| PPUBIRD1_2659 | Methylated-DNA--protein-cysteine methyltransferase | |  |  |
| PPUBIRD1_3233 | FAD dependent oxidoreductase | |  |  |
| PPUBIRD1_5067 | FAD dependent oxidoreductase | |  |  |
| PPUBIRD1_1102 | hypothetical protein | |  |  |
| PPUBIRD1_4946 | SerA | |  |  |
| PPUBIRD1_2079 | amino acid ABC transporter substrate-binding protein | |  |  |
| PPUBIRD1_1998 | Outer membrane porin | |  |  |
| PPUBIRD1_3230 | Deoxyribonuclease I | |  |  |
| PPUBIRD1_1126 | protein GlpF | |  |  |
| PPUBIRD1_2426 | TonB-dependent siderophore receptor | |  |  |
| PPUBIRD1_1977 | hypothetical protein | |  |  |
| PPUBIRD1_2581 | Aldehyde dehydrogenase family protein | |  |  |
| PPUBIRD1_1443 | Glutamate--putrescine ligase | |  |  |
| PPUBIRD1_3511 | LexA protein | |  |  |
| PPUBIRD1_2066 | decarboxylase | |  |  |
| PPUBIRD1_3085 | ABC transporter. permease/ATP-binding protein. putative | |  |  |
| PPUBIRD1_3229 | hypothetical protein | |  |  |
| PPUBIRD1_2524 | hypothetical protein | |  |  |
| PPUBIRD1_1429 | protein AlaS | |  |  |
| PPUBIRD1_2671 | hypothetical protein | |  |  |
| PPUBIRD1_2651 | Outer membrane autotransporter | |  |  |
| PPUBIRD1_0544 | Major facilitator family transporter | |  |  |
| PPUBIRD1_1752 | UvrC protein | |  |  |
| PPUBIRD1_4166 | hypothetical protein | |  |  |
| PPUBIRD1_2590 | Sugar transferase. putative | |  |  |
| PPUBIRD1_1837 | hypothetical protein | |  |  |
| PPUBIRD1_1873 | hypothetical protein | |  |  |
| PPUBIRD1_2953 | hypothetical protein | |  |  |
| PPUBIRD1_2751 | hypothetical protein | |  |  |
| PPUBIRD1_2391 | Curlin-associated protein | |  |  |
| PPUBIRD1_1814 | SerS protein | |  |  |
| PPUBIRD1_1649 | Electron transfer flavoprotein subunit beta | |  |  |
| PPUBIRD1_4038 | CspA protein | |  |  |
| PPUBIRD1_2144 | Flavin reductase domain-containing protein | |  |  |
| PPUBIRD1_0756 | Potassium efflux system protein | |  |  |
| PPUBIRD1_1689 | hypothetical protein | |  |  |
| PPUBIRD1_4870 | Type IV pili biogenesis protein | |  |  |
| PPUBIRD1_0402 | biotin biosynthesis protein BioC | |  |  |
| PPUBIRD1_4185 | 4-hydroxybenzoate transporter | |  |  |
| PPUBIRD1_0783 | hypothetical protein | |  |  |
| PPUBIRD1_1442 | BkdR | |  |  |
| PPUBIRD1_0687 | Fimbrial protein pilin | |  |  |
| PPUBIRD1_1105 | hypothetical protein | |  |  |
| PPUBIRD1_0796 | hypothetical protein | |  |  |
| PPUBIRD1_3398 | XRE family transcriptional regulator | |  |  |
| PPUBIRD1_1645 | hypothetical protein | |  |  |
| PPUBIRD1_t0055 | - | |  |  |
| PPUBIRD1_t0048 | - | |  |  |
| B GB DOWN. Common genes downregulated in cells grown in butanol as carbon source and glucose and butanol | | | | |
| PPUBIRD1_3513 | hypothetical protein | |  |  |
| PPUBIRD1_2747 | hypothetical protein | |  |  |
| PPUBIRD1_0882 | endoribonuclease L-PSP | |  |  |
| PPUBIRD1_4387 | HmuV | |  |  |
| PPUBIRD1_1991 | hypothetical protein | |  |  |
| PPUBIRD1_2773 | hypothetical protein | |  |  |
| PPUBIRD1_4312 | leucine dehydrogenase | |  |  |
| PPUBIRD1_1265 | Cation efflux protein | |  |  |
| PPUBIRD1_4500 | Putative type IV secretion system protein IcmJ/DotN | |  |  |
| PPUBIRD1_3985 | hypothetical protein | |  |  |
| PPUBIRD1_0926 | FAD dependent oxidoreductase | |  |  |
| PPUBIRD1_4170 | hypothetical protein | |  |  |
| PPUBIRD1_4239 | protein GmhA | |  |  |
| PPUBIRD1_4723 | hypothetical protein | |  |  |
| PPUBIRD1_0329 | Ricin B lectin | |  |  |
| PPUBIRD1_3805 | Lipopolysaccharide biosynthesis protein | |  |  |
| PPUBIRD1_4484 | hypothetical protein | |  |  |
| PPUBIRD1_4521 | hypothetical protein | |  |  |
| PPUBIRD1_0649 | Paraquat-inducible protein A | |  |  |
| PPUBIRD1_2742 | Putative ParB-like protein | |  |  |
| PPUBIRD1_0697 | gluconate transporter | |  |  |
| PPUBIRD1_1990 | Putative phage repressor | |  |  |
| PPUBIRD1_4869 | protein PilQ | |  |  |
| PPUBIRD1_4531 | Site-specific recombinase. phage integrase family domain protein | |  |  |
| PPUBIRD1_0806 | hypothetical protein | |  |  |
| PPUBIRD1_2764 | Major head protein | |  |  |
| PPUBIRD1_2766 | portal protein | |  |  |
| PPUBIRD1_3578 | ECF subfamily RNA polymerase sigma-24 factor | |  |  |
| PPUBIRD1_2765 | Peptidase S14 ClpP | |  |  |
| PPUBIRD1_2743 | Putative plasmid partitioning protein | |  |  |
| GB DOWN. Genes downregulated in cells grown in glucose and butanol | | |  |  |
| PPUBIRD1_2772 | Host specificity protein J | | | |
| PPUBIRD1_0842 | hypothetical protein | | | |
| PPUBIRD1_0722 | hypothetical protein | | | |
| PPUBIRD1_3929 | LysR family transcriptional regulator | | | |
| PPUBIRD1_1450 | protein CheR | | | |
| PPUBIRD1_0735 | hypothetical protein | | | |
| PPUBIRD1_0773 | hypothetical protein | | | |
| PPUBIRD1_4508 | Amino acid permease-associated region | | | |
| PPUBIRD1_0002 | transglycosylase | | | |
| PPUBIRD1_4889 | nucleoside-triphosphatase | | | |
| PPUBIRD1_3014 | hypothetical protein | | | |
| PPUBIRD1_3832 | hypothetical protein | | | |
| PPUBIRD1_2825 | GABA permease | | | |
| PPUBIRD1_1406 | LysR family transcriptional regulator | | | |
| PPUBIRD1_4511 | Major facilitator family transporter | | | |
| PPUBIRD1_3285 | hypothetical protein | | | |
| PPUBIRD1_0057 | protein GlmU | | | |
| PPUBIRD1_1593 | hypothetical protein | | | |
| PPUBIRD1_1845 | NAD-dependent epimerase/dehydratase | | | |
| PPUBIRD1_0639 | Bcr/CflA family multidrug resistance transporter | | | |
| PPUBIRD1_4523 | hypothetical protein | | | |
| PPUBIRD1_1345 | PhaJ1 | | | |
| PPUBIRD1_4532 | phage integrase family site-specific recombinase | | | |
| PPUBIRD1_2780 | IstB domain-containing protein ATP-binding protein | | | |
| PPUBIRD1_2748 | hypothetical protein | | | |
| PPUBIRD1_3732 | protein FadE | | | |
| PPUBIRD1_3661 | Two component LuxR family transcriptional regulator | | | |
| PPUBIRD1_0516 | protein RpoA | | | |
| PPUBIRD1_3796 | Alcohol dehydrogenase. zinc-containing | | | |
| PPUBIRD1_0691 | hypothetical protein | | | |
| PPUBIRD1_3757 | hypothetical protein | | | |
| PPUBIRD1_3803 | ABC transporter | | | |
| PPUBIRD1_3540 | methyl-accepting chemotaxis sensory transducer | | | |
| PPUBIRD1_0291 | Integral membrane sensor signal transduction histidine kinase | | | |
| PPUBIRD1_4640 | hypothetical protein | | | |
| PPUBIRD1_2835 | Acyl-homoserine lactone acylase pvdQ | | | |
| PPUBIRD1_2777 | Phage integrase family protein | | | |
| PPUBIRD1_4207 | AmpG-related permease | | | |
| PPUBIRD1_4825 | N-formimino-L-glutamate deiminase | | | |
| PPUBIRD1_0186 | Nicotinamide nucleotide transhydrogenase subunit alpha 1 | | | |
| PPUBIRD1_2502 | Protein maoC | | | |
| PPUBIRD1_4890 | Coproporphyrinogen III oxidase | | | |
| PPUBIRD1_1476 | N-acetyl neuramic acid synthetase NeuB | | | |
| PPUBIRD1_3541 | Pseudouridine synthase | | | |
| PPUBIRD1_3915 | RdgC | | | |
| PPUBIRD1_2868 | Pyridine nucleotide-disulfide oxidoreductase family protein | | | |
| PPUBIRD1_0594 | Aldehyde dehydrogenase | | | |
| PPUBIRD1_1468 | protein FliS | | | |
| PPUBIRD1_3247 | aminotransferase. class V | | | |
| PPUBIRD1_2746 | Prophage PSPPH02. adenine modification methytransferase | | | |
| PPUBIRD1_0190 | TonB-dependent siderophore receptor | | | |
| PPUBIRD1_4790 | hypothetical protein | | | |
| PPUBIRD1_2810 | Mqo3 | | | |
| PPUBIRD1_2131 | Permease for cytosine/purine. uracil. thiamine. allantoin | | | |
| PPUBIRD1_2789 | hypothetical protein | | | |
| PPUBIRD1_0627 | hypothetical protein | | | |
| PPUBIRD1_0820 | Pta | | | |
| PPUBIRD1_0766 | protein Pth | | | |
| PPUBIRD1_0148 | Periplasmic solute binding protein | | | |
| PPUBIRD1_3067 | hypothetical protein | | | |
| PPUBIRD1_0024 | Sodium/hydrogen exchanger | | | |
| PPUBIRD1_0512 | hypothetical protein | | | |
| GB S DOWN. Common genes downregulated in cells grown in glucose and butanol and in cells after a butanol shock | | | | |
| PPUBIRD1_3867 | Carbon storage regulator. CsrA | | | |
| PPUBIRD1_4306 | hypothetical protein | | | |
| PPUBIRD1_1079 | hypothetical protein | | | |
| PPUBIRD1_1395 | Spy-related protein | | | |
| PPUBIRD1_0753 | hypothetical protein | | | |
| PPUBIRD1_2373 | Carbohydrate kinase | | | |
| PPUBIRD1_1551 | Major facilitator transporter | | | |
| PPUBIRD1_4050 | hypothetical protein | | | |
| PPUBIRD1_4726 | Glycosyl transferase. putative | | | |
| PPUBIRD1_1458 | protein FlgH | | | |
| PPUBIRD1_3983 | hypothetical protein | | | |
| PPUBIRD1_4939 | hypothetical protein | | | |
| PPUBIRD1_1989 | hypothetical protein | | | |
| PPUBIRD1_4440 | D-lactate dehydrogenase | | | |
| PPUBIRD1_4581 | Lytic murein transglycosylase | | | |
| PPUBIRD1_3333 | Multi-sensor hybrid histidine kinase | | | |
| PPUBIRD1_4588 | protein MltB | | | |
| B GB S DOWN. Common genes downregulated in the three conditions | | | | |
| PPUBIRD1_t0033 |  | | | |
| PPUBIRD1_2078 | TetR family transcriptional regulator | | | |
| PPUBIRD1_4148 | hypothetical protein | | | |
| PPUBIRD1_0460 | hypothetical protein | | | |
| PPUBIRD1_3231 | hypothetical protein | | | |
| PPUBIRD1_1433 | AlgZ protein | | | |
| PPUBIRD1_4662 | hypothetical protein | | | |
| PPUBIRD1_4844 | protein Pgm | | | |
| PPUBIRD1_4149 | Pseudouridine synthase | | | |
| PPUBIRD1_4236 | Uroporphyrin-III C/tetrapyrrole methyltransferase | | | |
| GB S UP. Common genes upregulated in cells grown in glucose and butanol and in cells after a butanol shock | | | | |
| PPUBIRD1_1249 | hypothetical protein | | | |
| PPUBIRD1_1334 | Putative lipoprotein | | | |
