# Supplementary material for: Understanding butanol tolerance and assimilation in P seudomonas putida BIRD‐1: an integrated omics approach
Source: Microb Biotechnol. 2016 Jan 6;9(1):100–15. doi: 10.1111/1751-7915.12328 (PMC4720416; doi:10.1111/1751-7915.12328)
Supplement: Supplementary file 1 — Fig. S1. Cell death kinetics after a butanol shock of BIRD‐1, KT2440 and DOT‐T1E. Killing kinetics of P. putida strains upon exposure to different butanol concentrations. The strains were grown to reach the exponential phase (turbidity of 0.85 at 660 nm). At t = 0 the culture was divided into two aliquots, to which 1% or 2% (v/v) butanol was added. At the indicated times, the number of viable cells were estimated by plating dilutions on LB. Fig. S2. ppGpp response model. ppGpp accumulation is mediated by the SpoT protein. In the genome, spoT is located downstream of rpoZ, which is the omega subunit of RNA polymerase. Table S1. Doubling time of P. putida BIRD‐1, KT2440 and DOT‐T1E growing on different media. Doubling times (G) and lag phases (lag) are indicated. Table S2. Mutant library characteristics and phenotypes. Mutants in a mutant library, insertion points of the sequences obtained and phenotype (A, assimilation, T, tolerance and A&T, assimilation and tolerance). Table S3. Venn Diagram specification. Butanol as sole carbon source, Shock and glucose butanol grown cells. Each transcript found in common in the diagram is categorized. Table S4. Transcriptomics results. Table obtained after comparison of the all the conditions versus the control (glucose grown cells). Table S5. Peptides whole cell proteome detected by MS/MS List of redundant peptides obtained from whole cell proteome of the three biological replicates of the control (C), butanol grown cells (B), glucose plus butanol grown cells (GB) and cells after a butanol shock (S). Table S6. Pattern Lab analysis of whole cell proteome. List of proteins from whole cells of P. putida BIRD‐1 validated with at least two different peptides. Table S7. Peptides membrane proteome detected by MS/MS. List of redundant peptides obtained from membrane proteins of the three biological replicates of the control (C), butanol grown cells (B), glucose plus butanol grown cells (GB) and cells after a butanol shock (S). Table S8. [file MBT2-9-100-s001.zip › TableS4.docx]

**Table S4**

| **Butanol 0.3%** |  |  |  |
| --- | --- | --- | --- |
| **Synonym** | **Product** | **Fold change** | **p-value** |
| **TCA cycle and related proteins** | | | |
| PPUBIRD1_2615 | Aldo/keto reductase (gluconate related) | 30,5 | 0,002 |
| PPUBIRD1_2374 | LacI family transcriptional regulator (gluconate) | 19 | 0,000 |
| PPUBIRD1_2223 | Acetylornithine deacetylase | 9,5 | 0,003 |
| PPUBIRD1_4941 | RpiA (carbon metabolism) | 7,77 | 0,010 |
| PPUBIRD1_0531 | Formate dehydrogenase accessory protein FdhE | 7,33 | 0,003 |
| PPUBIRD1_2372 | GntP protein gluconate transporter | 5,71 | 0,005 |
| PPUBIRD1_1842 | PcaI (acetyl-coA) | 3,89 | 0,020 |
| PPUBIRD1_1803 | isocitrate dehydrogenase | 3.60 | 0.015 |
| PPUBIRD1_1985 | L-ornithine N5-oxygenase | 3.48 | 0.002 |
| PPUBIRD1_3075 | Fumarate reductase/succinate dehydrogenase flavoprotein domain protein | 3.31 | 0.007 |
| PPUBIRD1_3877 | Beta (1-6) glucans synthase. putative (carbohydrate) | 2.4 | 0.011 |
| PPUBIRD1_2140 | Aldehyde dehydrogenase | 2.39 | 0.015 |
| PPUBIRD1_4171 | Oxaloacetate decarboxylase (arginine metabolism) | 2.22 | 0.010 |
| PPUBIRD1_4315 | Fumarylacetoacetase | -2.26 | 0.001 |
| PPUBIRD1_2404 | gluconate 2-dehydrogenase | -2.65 | 0.001 |
| PPUBIRD1_3791 | glutathione S-transferase | -2.78 | 0.002 |
| PPUBIRD1_1110 | glutamate synthase (NADPH) | -2.97 | 0.013 |
| PPUBIRD1_4844 | protein Pgm (phosphoglyceromutase) | -3 | 0.012 |
| PPUBIRD1_0697 | gluconate transporter | -3.56 | 0.010 |
| PPUBIRD1_1131 | Glutaredoxin-like protein | -6.17 | 0.012 |
| PPUBIRD1_1422 | AruF (arginine ornithine) | -7.08 | 0.013 |
| PPUBIRD1_1071 | DNA-binding transcriptional regulator HexR (glucose-gluconate-ketogluconate) | -57.47 | 0.000 |
| **Efflux pumps and resistance proteins** | | | |
| PPUBIRD1_3000 | Extracellular solute-binding protein | 111.00 | 0.018 |
| PPUBIRD1_4325 | MerR family transcriptional regulator (mercuric resistance operon) | 16.68 | 0.000 |
| PPUBIRD1_2317 | Type II secretion system protein G | 3 | 0.000 |
| PPUBIRD1_2362 | MexF | 2.98 | 0.008 |
| PPUBIRD1_0759 | Secretion protein HlyD family protein | 2.94 | 0.020 |
| PPUBIRD1_3167 | Outer membrane porin | 2.38 | 0.013 |
| PPUBIRD1_2631 | Major facilitator transporter | 2.24 | 0.011 |
| PPUBIRD1_1850 | Extracellular solute-binding protein | 2.05 | 0.007 |
| PPUBIRD1_0758 | NodT family RND efflux system outer membrane lipoprotein | -3.04 | 0.004 |
| PPUBIRD1_4869 | protein PilQ (type II or IV) | -3.12 | 0.007 |
| PPUBIRD1_3806 | Polysaccharide export protein | -3.51 | 0.004 |
| PPUBIRD1_1548 | mechanosensitive ion channel protein MscS | -3.54 | 0.001 |
| PPUBIRD1_4505 | Putative type IV secretion system protein IcmK/DotH | -5.67 | 0.001 |
| PPUBIRD1_4500 | Putative type IV secretion system protein IcmJ/DotN | -8.14 | 0.003 |
| PPUBIRD1_1265 | Cation efflux protein | -9.64 | 0.003 |
| PPUBIRD1_4502 | Putative type IV secretion system protein IcmC/DotIE | -12.67 | 0.010 |
| PPUBIRD1_2078 | TetR family transcriptional regulator | -29.00 | 0.005 |
| **Lipid metabolism** | | | |
| PPUBIRD1_2478 | Lipoprotein OprI. putative | 207.50 | 0.000 |
| PPUBIRD1_0399 | protein BioB (biotin synthase) | 5.50 | 0.008 |
| PPUBIRD1_2470 | protein MalK (lypopolysacharide biosynthesis) | 4.36 | 0.006 |
| PPUBIRD1_3532 | Putative lipoprotein | 4.01 | 0.003 |
| PPUBIRD1_0240 | Fatty acid desaturase | -2.14 | 0.001 |
| PPUBIRD1_3766 | Enoyl-CoA hydratase (lipid) | -3.50 | 0.010 |
| PPUBIRD1_4516 | Acyl-CoA thioesterase II (fatty acids) | -3.52 | 0.008 |
| PPUBIRD1_3805 | Lipopolysaccharide biosynthesis protein | -4.07 | 0.007 |
| PPUBIRD1_4239 | protein GmhA (phosphoheptose isomerase) | -5.61 | 0.008 |
| PPUBIRD1_3810 | protein KdsA | -6.79 | 0.005 |
| PPUBIRD1_4011 | protein LpxB | -13.00 | 0.009 |
| PPUBIRD1_3437 | FadB2 | -41.00 | 0.016 |
| **Ferric related proteins** | | | |
| PPUBIRD1_2952 | hemerythrin HHE cation binding domain-containing protein | 122.50 | 0.014 |
| PPUBIRD1_2177 | TonB-dependent siderophore receptor | 7.41 | 0.006 |
| PPUBIRD1_3261 | Anti-FecI sigma factor. FecR | 4.49 | 0.016 |
| PPUBIRD1_1681 | TonB-dependent receptor. plug | 2.38 | 0.020 |
| PPUBIRD1_3580 | Ferric-pseudobactin M114 receptor pbuA | 2.38 | 0.019 |
| PPUBIRD1_3497 | Heavy metal sensor signal transduction histidine kinase | -3.08 | 0.010 |
| PPUBIRD1_4387 | HmuV | -39.50 | 0.004 |
| **Energy production** | | | |
| PPUBIRD1_1728 | NADH dehydrogenase subunit E (quinone oxidoreductase) | 45.50 | 0.001 |
| PPUBIRD1_1600 | CcoO (cytochrome c oxidase) | 22.50 | 0.011 |
| PPUBIRD1_3002 | QedH (PQQ-cytochrome c) | 14.50 | 0.013 |
| PPUBIRD1_1526 | protein CcmC (cytochrome c related) | 7.50 | 0.004 |
| PPUBIRD1_2849 | Cytochrome B561 | 4.50 | 0.015 |
| PPUBIRD1_0340 | Oxidoreductase. FMN-binding protein | -4.41 | 0.008 |
| **Cell division** | | | |
| PPUBIRD1_3883 | protein MinC (septum formation inhibitor) | 3.32 | 0.014 |
| PPUBIRD1_2743 | Putative plasmid partitioning protein | -2.06 | 0.018 |
| PPUBIRD1_2742 | Putative ParB-like protein | -3.60 | 0.017 |
| PPUBIRD1_4548 | ATP-dependent helicase HrpB | -5.38 | 0.001 |
| PPUBIRD1_3835 | Glycosyltransferases involved in cell wall biogenesis | -6.26 | 0.016 |
| PPUBIRD1_4233 | cell division protein FtsL | -10.75 | 0.016 |
| **Transcriptional regulators** | | | |
| PPUBIRD1_3004 | Two component LuxR family transcriptional regulator | 35.13 | 0.003 |
| PPUBIRD1_2619 | LexA repressor | 18.00 | 0.003 |
| PPUBIRD1_2108 | Transcriptional regulator MvaT. P16 subunit. putative | 14.50 | 0.011 |
| PPUBIRD1_3011 | Two component LuxR family transcriptional regulator | 12.73 | 0.005 |
| PPUBIRD1_2589 | LysR family transcriptional regulator | 6.62 | 0.009 |
| PPUBIRD1_2189 | GntR family transcriptional regulator | 3.65 | 0.019 |
| PPUBIRD1_2063 | AraC family transcriptional regulator | 2.27 | 0.006 |
| PPUBIRD1_3684 | LysR family transcriptional regulator | 2.05 | 0.002 |
| PPUBIRD1_3395 | GAF modulated Fis family sigma-54 specific transcriptional regulator | -2.70 | 0.009 |
| PPUBIRD1_0041 | LysR family transcriptional regulator | -3.51 | 0.007 |
| PPUBIRD1_1433 | AlgZ protein (alginate production) | -7.66 | 0.003 |
| PPUBIRD1_1062 | GltR_2 | -16.86 | 0.012 |
| PPUBIRD1_2902 | LysR family transcriptional regulator | -17.60 | 0.004 |
| **Diguanylate cyclase related proteins** | | | |
| PPUBIRD1_3396 | Diguanylate cyclase/phosphodiesterase with PAS/PAC and GAF sensor(s) | 9.52 | 0.007 |
| PPUBIRD1_2211 | signaling protein (diguanylate cyclase) | 7.58 | 0.012 |
| PPUBIRD1_0447 | PAS/PAC sensor signal transduction histidine kinase (diguanylate cyclase) | -2.20 | 0.014 |
| **tRNA related proteins** | | | |
| PPUBIRD1_t0026 | Leu tRNA (Aminoacyl-tRNA biosynthesis) | 99.50 | 0.001 |
| PPUBIRD1_1808 | Putative arginyl-tRNA--protein transferase | 3.73 | 0.006 |
| PPUBIRD1_3463 | TRNA--hydroxylase | 2.56 | 0.014 |
| PPUBIRD1_t0033 | Ser tRNA | -257.50 | 0.000 |
| **Hypothetical proteins** | | | |
| PPUBIRD1_2386 | hypothetical protein | 290.00 | 0.013 |
| PPUBIRD1_1170 | hypothetical protein | 149.50 | 0.017 |
| PPUBIRD1_3341 | hypothetical protein | 64.00 | 0.019 |
| PPUBIRD1_2179 | hypothetical protein | 42.50 | 0.011 |
| PPUBIRD1_2332 | hypothetical protein | 31.50 | 0.020 |
| PPUBIRD1_2350 | hypothetical protein | 29.00 | 0.001 |
| PPUBIRD1_4681 | hypothetical protein | 22.50 | 0.000 |
| PPUBIRD1_0130 | hypothetical protein | 17.18 | 0.000 |
| PPUBIRD1_2180 | hypothetical protein | 10.86 | 0.005 |
| PPUBIRD1_3216 | hypothetical protein | 8.68 | 0.018 |
| PPUBIRD1_4947 | hypothetical protein | 8.22 | 0.001 |
| PPUBIRD1_2678 | hypothetical protein | 5.33 | 0.013 |
| PPUBIRD1_2878 | hypothetical protein | 5.01 | 0.012 |
| PPUBIRD1_2292 | hypothetical protein | 4.49 | 0.005 |
| PPUBIRD1_3101 | hypothetical protein | 4.36 | 0.018 |
| PPUBIRD1_3376 | hypothetical protein | 4.29 | 0.003 |
| PPUBIRD1_2983 | hypothetical protein | 4.01 | 0.002 |
| PPUBIRD1_1521 | hypothetical protein | 3.23 | 0.008 |
| PPUBIRD1_2749 | hypothetical protein | 3.16 | 0.008 |
| PPUBIRD1_2286 | hypothetical protein | 3.14 | 0.015 |
| PPUBIRD1_1955 | hypothetical protein | 2.96 | 0.008 |
| PPUBIRD1_0964 | hypothetical protein | 2.88 | 0.001 |
| PPUBIRD1_2186 | hypothetical protein | 2.85 | 0.014 |
| PPUBIRD1_3305 | hypothetical protein | 2.73 | 0.004 |
| PPUBIRD1_1878 | hypothetical protein | 2.46 | 0.019 |
| PPUBIRD1_3959 | hypothetical protein | 2.24 | 0.009 |
| PPUBIRD1_4272 | hypothetical protein | 2.08 | 0.012 |
| PPUBIRD1_1388 | hypothetical protein | -2.50 | 0.005 |
| PPUBIRD1_1993 | hypothetical protein | -2.53 | 0.017 |
| PPUBIRD1_3980 | hypothetical protein | -2.53 | 0.004 |
| PPUBIRD1_3667 | hypothetical protein | -2.59 | 0.005 |
| PPUBIRD1_3798 | hypothetical protein | -2.69 | 0.002 |
| PPUBIRD1_0806 | hypothetical protein | -2.79 | 0.001 |
| PPUBIRD1_2794 | hypothetical protein | -2.83 | 0.006 |
| PPUBIRD1_3718 | hypothetical protein | -2.88 | 0.008 |
| PPUBIRD1_0832 | hypothetical protein | -3.31 | 0.006 |
| PPUBIRD1_0539 | hypothetical protein | -3.70 | 0.016 |
| PPUBIRD1_4521 | hypothetical protein | -3.86 | 0.010 |
| PPUBIRD1_4484 | hypothetical protein | -3.98 | 0.005 |
| PPUBIRD1_2795 | hypothetical protein | -4.03 | 0.009 |
| PPUBIRD1_4662 | hypothetical protein | -4.50 | 0.004 |
| PPUBIRD1_4547 | hypothetical protein | -4.89 | 0.016 |
| PPUBIRD1_0581 | hypothetical protein | -4.89 | 0.010 |
| PPUBIRD1_3386 | hypothetical protein | -4.89 | 0.010 |
| PPUBIRD1_5086 | hypothetical protein | -5.25 | 0.010 |
| PPUBIRD1_4723 | hypothetical protein | -5.59 | 0.015 |
| PPUBIRD1_4170 | hypothetical protein | -5.90 | 0.002 |
| PPUBIRD1_3985 | hypothetical protein | -6.32 | 0.018 |
| PPUBIRD1_1942 | hypothetical protein | -6.33 | 0.015 |
| PPUBIRD1_5087 | hypothetical protein | -6.46 | 0.011 |
| PPUBIRD1_1221 | hypothetical protein | -6.93 | 0.003 |
| PPUBIRD1_3231 | hypothetical protein | -8.30 | 0.003 |
| PPUBIRD1_0460 | hypothetical protein | -9.38 | 0.019 |
| PPUBIRD1_4148 | hypothetical protein | -10.15 | 0.009 |
| PPUBIRD1_1824 | hypothetical protein | -10.30 | 0.003 |
| PPUBIRD1_1330 | hypothetical protein | -12.75 | 0.018 |
| PPUBIRD1_2773 | hypothetical protein | -14.86 | 0.000 |
| PPUBIRD1_4920 | hypothetical protein | -15.60 | 0.005 |
| PPUBIRD1_2761 | hypothetical protein | -22.50 | 0.003 |
| PPUBIRD1_1991 | hypothetical protein | -36.50 | 0.015 |
| PPUBIRD1_2747 | hypothetical protein | -45.00 | 0.017 |
| PPUBIRD1_3513 | hypothetical protein | -56.07 | 0.002 |
| PPUBIRD1_1482 | hypothetical protein | -85.00 | 0.011 |
| **Unclassified proteins** | | | |
| PPUBIRD1_2647 | BdhA (hydroxybutyrate - butanoate metabolism) | 44.00 | 0.001 |
| PPUBIRD1_1001 | PtsO (nitrogen regulation) | 32.10 | 0.001 |
| PPUBIRD1_2235 | binding-protein-dependent transport system inner membrane protein | 29.00 | 0.005 |
| PPUBIRD1_0117 | OsmC family protein (osmotically induced protein) | 25.00 | 0.006 |
| PPUBIRD1_3045 | AmiS/UreI transporter | 24.50 | 0.010 |
| PPUBIRD1_3003 | Pentapeptide repeat-containing protein | 18.56 | 0.009 |
| PPUBIRD1_2487 | PhaM (phenylacetic acid degradation protein) | 18.50 | 0.001 |
| PPUBIRD1_2990 | D-serine dehydratase | 13.65 | 0.011 |
| PPUBIRD1_2931 | Acetyltransferase | 9.95 | 0.012 |
| PPUBIRD1_3374 | TatD-related deoxyribonuclease (hydrolase) | 8.00 | 0.020 |
| PPUBIRD1_2501 | PhaK (putative phenylacetic acid-specific porin PhaK) | 4.48 | 0.004 |
| PPUBIRD1_2043 | Periplasmic polyamine-binding protein. putative (putrescine/spermidine transporter) | 4.00 | 0.005 |
| PPUBIRD1_1326 | AAA ATPase | 3.82 | 0.006 |
| PPUBIRD1_3864 | Acetyltransferase (cyanophycin synthase) | 3.79 | 0.011 |
| PPUBIRD1_3903 | Peptidylprolyl isomerase FKBP-type | 3.41 | 0.014 |
| PPUBIRD1_2848 | Catalase domain protein (inorganic transport and metabolism) | 3.28 | 0.011 |
| PPUBIRD1_0286 | HAD family hydrolase | 3.26 | 0.014 |
| PPUBIRD1_3375 | methyl-accepting chemotaxis sensory transducer | 3.15 | 0.009 |
| PPUBIRD1_3007 | YVTN family beta-propeller repeat-containing protein | 2.97 | 0.007 |
| PPUBIRD1_3897 | Alcohol dehydrogenase. zinc-containing (quinone reductase) | 2.94 | 0.011 |
| PPUBIRD1_1541 | Hydantoin racemase. putative (Asp/Glu/Hydantoin racemase) | 2.93 | 0.004 |
| PPUBIRD1_4795 | protein PhaF (multicomponent K+:H+ antiporter subunit F) | 2.84 | 0.009 |
| PPUBIRD1_1827 | short-chain dehydrogenase | 2.74 | 0.020 |
| PPUBIRD1_2673 | Alcohol dehydrogenase (quinone reductase) | 2.71 | 0.016 |
| PPUBIRD1_1963 | binding-protein-dependent transport system inner membrane protein | 2.35 | 0.000 |
| PPUBIRD1_0471 | anhydro-N-acetylmuramic acid kinase | 2.27 | 0.000 |
| PPUBIRD1_3556 | RlmL (23S rRNA (guanine)-methyltransferase) | 2.12 | 0.018 |
| PPUBIRD1_2640 | Phospho-2-dehydro-3-deoxyheptonate aldolase (phenylalanine. tyrosine. tryptophan) | 2.05 | 0.017 |
| PPUBIRD1_1179 | FAD dependent oxidoreductase | -2.03 | 0.001 |
| PPUBIRD1_4236 | Uroporphyrin-III C/tetrapyrrole methyltransferase | -2.13 | 0.012 |
| PPUBIRD1_3977 | Protein sprT | -2.15 | 0.000 |
| PPUBIRD1_1150 | Dcd (Pyrimidine metabolism) | -2.29 | 0.019 |
| PPUBIRD1_4151 | PhaG (multicomponent K+:H+ antiporter subunit G) | -2.30 | 0.017 |
| PPUBIRD1_2765 | Peptidase S14 ClpP | -2.34 | 0.014 |
| PPUBIRD1_4149 | Pseudouridine synthase | -2.38 | 0.016 |
| PPUBIRD1_3578 | ECF subfamily RNA polymerase sigma-24 factor | -2.42 | 0.013 |
| PPUBIRD1_2766 | portal protein | -2.49 | 0.020 |
| PPUBIRD1_0944 | Intracellular protease. PfpI family | -2.50 | 0.011 |
| PPUBIRD1_2764 | Major head protein | -2.65 | 0.008 |
| PPUBIRD1_1917 | Lambda family phage tail tape measure protein | -2.75 | 0.000 |
| PPUBIRD1_1246 | Cold-shock DNA-binding domain-containing protein | -2.79 | 0.015 |
| PPUBIRD1_4068 | Putative CheW protein (chemotaxis) | -2.81 | 0.016 |
| PPUBIRD1_4531 | Site-specific recombinase. phage integrase family domain protein | -3.03 | 0.020 |
| PPUBIRD1_4916 | Putative signal transduction protein | -3.38 | 0.017 |
| PPUBIRD1_1990 | Putative phage repressor | -3.40 | 0.002 |
| PPUBIRD1_0649 | Paraquat-inducible protein A | -3.80 | 0.017 |
| PPUBIRD1_3520 | Universal stress protein | -4.06 | 0.004 |
| PPUBIRD1_0909 | Putative aminotransferase | -4.17 | 0.006 |
| PPUBIRD1_0329 | Ricin B lectin | -4.27 | 0.015 |
| PPUBIRD1_0311 | GabP (aminoacid) GABA permease | -4.53 | 0.014 |
| PPUBIRD1_0051 | Histidine kinase | -4.59 | 0.001 |
| PPUBIRD1_0326 | Sda (serine dehidratase) | -5.00 | 0.006 |
| PPUBIRD1_0926 | FAD dependent oxidoreductase | -6.15 | 0.002 |
| PPUBIRD1_1583 | Major facilitator family transporter | -8.00 | 0.000 |
| PPUBIRD1_2405 | EndA (endonuclease) | -8.71 | 0.009 |
| PPUBIRD1_1286 | Amino acid transporter LysE | -9.09 | 0.013 |
| PPUBIRD1_4312 | leucine dehydrogenase (Valine. leucine and isoleucine degradation) | -10.23 | 0.006 |
| PPUBIRD1_2685 | AroE_2 (shikimate - phenilalanine. tryptophan metabolism) | -14.00 | 0.020 |
| PPUBIRD1_0693 | ISPsy5. Orf1 | -29.00 | 0.005 |
| PPUBIRD1_0882 | endoribonuclease L-PSP | -40.36 | 0.001 |

| **Glucosa+butanol 0.3%** | |  |  |
| --- | --- | --- | --- |
| **Synonym** | **Product** | **Fold change** | **p-value** |
| **TCA cycle and related proteins** | | | |
| PPUBIRD1_1842 | PcaI (acetyl-coA- butanoate. propanoate metabolism) | 5.36 | 0.016 |
| PPUBIRD1_2490 | protein PaaC (3-hydroxyacyl-CoA dehydrogenase- phenylalanine metabolism) | 2.83 | 0.008 |
| PPUBIRD1_1443 | Glutamate--putrescine ligase | 2.77 | 0.013 |
| PPUBIRD1_2279 | 5-oxoprolinase (glutathione metabolism) | 2.16 | 0.009 |
| PPUBIRD1_0820 | Pta (phosphate acetyltransferase) | -2.26 | 0.005 |
| PPUBIRD1_0697 | gluconate transporter | -2.54 | 0.011 |
| PPUBIRD1_0594 | Aldehyde dehydrogenase (glycolisis) | -2.77 | 0.002 |
| PPUBIRD1_1476 | N-acetyl neuramic acid synthetase NeuB (sugar metabolism) | -3.26 | 0.018 |
| PPUBIRD1_4844 | protein Pgm (phosphoglyceromutase) | -3.27 | 0.016 |
| PPUBIRD1_4825 | N-formimino-L-glutamate deiminase | -3.58 | 0.008 |
| PPUBIRD1_0057 | protein GlmU (glucosamine-1-phosphate N-acetyltransferase - sugar metabolism) | -19.00 | 0.018 |
| PPUBIRD1_2373 | Carbohydrate kinase (gluconate kinase) | -27.00 | 0.001 |
| **Efflux pumps and resistance proteins** | | | |
| PPUBIRD1_4870 | Type IV pili biogenesis protein | 13.00 | 0.006 |
| PPUBIRD1_0756 | Potassium efflux system protein | 11.50 | 0.002 |
| PPUBIRD1_0544 | Major facilitator family transporter | 3.49 | 0.020 |
| PPUBIRD1_2651 | Outer membrane autotransporter | 3.38 | 0.005 |
| PPUBIRD1_1998 | Outer membrane porin | 2.58 | 0.006 |
| PPUBIRD1_4869 | protein PilQ (type II or IV) | -5.81 | 0.008 |
| PPUBIRD1_1265 | Cation efflux protein | -6.45 | 0.005 |
| PPUBIRD1_4500 | Putative type IV secretion system protein IcmJ/DotN | -11.19 | 0.010 |
| PPUBIRD1_0639 | Bcr/CflA family multidrug resistance transporter | -11.50 | 0.011 |
| PPUBIRD1_2078 | TetR family transcriptional regulator | -29.00 | 0.005 |
| **Lipid metabolism** | | | |
| PPUBIRD1_1334 | Putative lipoprotein | 41.50 | 0.012 |
| PPUBIRD1_0402 | biotin biosynthesis protein BioC (biotin synthase) | 17.00 | 0.003 |
| PPUBIRD1_0399 | protein BioB (biotin synthase) | 14.00 | 0.020 |
| PPUBIRD1_3805 | Lipopolysaccharide biosynthesis protein | -2.85 | 0.002 |
| PPUBIRD1_3732 | protein FadE (acyl-CoA dehydrogenase) | -6.57 | 0.015 |
| PPUBIRD1_4239 | protein GmhA (phosphoheptose isomerase) | -10.65 | 0.012 |
| **Ferric related proteins** | | | |
| PPUBIRD1_2426 | TonB-dependent siderophore receptor | 2.69 | 0.004 |
| PPUBIRD1_4387 | HmuV | -2.19 | 0.015 |
| PPUBIRD1_0190 | TonB-dependent siderophore receptor | -2.57 | 0.019 |
| **Energy production** | | | |
| PPUBIRD1_1649 | Electron transfer flavoprotein subunit beta | 5.52 | 0.013 |
| PPUBIRD1_1958 | Cytochrome c. class I | 2.24 | 0.001 |
| PPUBIRD1_4890 | Coproporphyrinogen III oxidase | -3.35 | 0.014 |
| **Cell division** | | | |
| PPUBIRD1_2742 | Putative ParB-like protein (chromosome partitioning protein) | -4.62 | 0.017 |
| PPUBIRD1_2743 | Putative plasmid partitioning protein | -17.33 | 0.009 |
| **Transcriptional regulators** | | | |
| PPUBIRD1_3398 | XRE family transcriptional regulator | 46.50 | 0.009 |
| PPUBIRD1_2189 | GntR family transcriptional regulator | 3.52 | 0.003 |
| PPUBIRD1_3511 | LexA protein | 2.80 | 0.009 |
| PPUBIRD1_3028 | LysR family transcriptional regulator | 2.33 | 0.001 |
| PPUBIRD1_3661 | Two component LuxR family transcriptional regulator | -6.10 | 0.010 |
| PPUBIRD1_1406 | LysR family transcriptional regulator | -21.50 | 0.005 |
| PPUBIRD1_3929 | LysR family transcriptional regulator | -89.00 | 0.009 |
| PPUBIRD1_1433 | AlgZ protein (alginate production) | -234.00 | 0.001 |
| **tRNA related proteins** | | | |
| PPUBIRD1_t0048 | His tRNA (Aminoacyl-tRNA biosynthesis) | 126.50 | 0.013 |
| PPUBIRD1_t0055 | Leu tRNA (Aminoacyl-tRNA biosynthesis) | 110.00 | 0.001 |
| PPUBIRD1_1814 | SerS protein (seryl-tRNA synthetase) | 4.83 | 0.009 |
| PPUBIRD1_1429 | protein AlaS (Alanyl-tRNA synthetase) | 3.14 | 0.000 |
| PPUBIRD1_0766 | protein Pth (Peptidyl-tRNA hydrolase) | -2.24 | 0.016 |
| PPUBIRD1_t0033 | Ser tRNA (Aminoacyl-tRNA biosynthesis) | -257.50 | 0.000 |
| **Hypothetical proteins** | | | |
| PPUBIRD1_1645 | hypothetical protein | 49.50 | 0.003 |
| PPUBIRD1_0796 | hypothetical protein | 42.50 | 0.011 |
| PPUBIRD1_1105 | hypothetical protein | 35.50 | 0.010 |
| PPUBIRD1_0783 | hypothetical protein | 25.50 | 0.009 |
| PPUBIRD1_1249 | hypothetical protein | 20.33 | 0.003 |
| PPUBIRD1_1689 | hypothetical protein | 12.50 | 0.014 |
| PPUBIRD1_3216 | hypothetical protein | 11.53 | 0.013 |
| PPUBIRD1_1878 | hypothetical protein | 8.11 | 0.000 |
| PPUBIRD1_4947 | hypothetical protein | 5.78 | 0.009 |
| PPUBIRD1_2983 | hypothetical protein | 4.49 | 0.008 |
| PPUBIRD1_2751 | hypothetical protein | 4.27 | 0.007 |
| PPUBIRD1_2953 | hypothetical protein | 4.16 | 0.016 |
| PPUBIRD1_1873 | hypothetical protein | 4.16 | 0.011 |
| PPUBIRD1_1837 | hypothetical protein | 4.11 | 0.014 |
| PPUBIRD1_4166 | hypothetical protein | 3.51 | 0.015 |
| PPUBIRD1_2671 | hypothetical protein | 3.25 | 0.013 |
| PPUBIRD1_2524 | hypothetical protein | 3.13 | 0.002 |
| PPUBIRD1_3229 | hypothetical protein | 3.13 | 0.008 |
| PPUBIRD1_1977 | hypothetical protein | 2.71 | 0.017 |
| PPUBIRD1_1102 | hypothetical protein | 2.43 | 0.003 |
| PPUBIRD1_3822 | hypothetical protein | 2.26 | 0.017 |
| PPUBIRD1_2231 | hypothetical protein | 2.08 | 0.009 |
| PPUBIRD1_4467 | hypothetical protein | 2.04 | 0.012 |
| PPUBIRD1_0512 | hypothetical protein | -2.01 | 0.013 |
| PPUBIRD1_4170 | hypothetical protein | -2.13 | 0.007 |
| PPUBIRD1_4484 | hypothetical protein | -2.13 | 0.008 |
| PPUBIRD1_3067 | hypothetical protein | -2.14 | 0.008 |
| PPUBIRD1_0627 | hypothetical protein | -2.28 | 0.019 |
| PPUBIRD1_2789 | hypothetical protein | -2.31 | 0.016 |
| PPUBIRD1_4662 | hypothetical protein | -2.39 | 0.012 |
| PPUBIRD1_4790 | hypothetical protein | -2.55 | 0.008 |
| PPUBIRD1_1989 | hypothetical protein | -2.71 | 0.006 |
| PPUBIRD1_4521 | hypothetical protein | -3.00 | 0.019 |
| PPUBIRD1_4939 | hypothetical protein | -3.65 | 0.005 |
| PPUBIRD1_3983 | hypothetical protein | -3.86 | 0.008 |
| PPUBIRD1_0806 | hypothetical protein | -4.38 | 0.000 |
| PPUBIRD1_4640 | hypothetical protein | -4.89 | 0.016 |
| PPUBIRD1_3757 | hypothetical protein | -5.39 | 0.019 |
| PPUBIRD1_0691 | hypothetical protein | -5.50 | 0.008 |
| PPUBIRD1_2773 | hypothetical protein | -5.75 | 0.000 |
| PPUBIRD1_4050 | hypothetical protein | -6.04 | 0.003 |
| PPUBIRD1_3513 | hypothetical protein | -7.08 | 0.014 |
| PPUBIRD1_2748 | hypothetical protein | -7.57 | 0.005 |
| PPUBIRD1_4523 | hypothetical protein | -11.00 | 0.000 |
| PPUBIRD1_0130 | hypothetical protein | -11.20 | 0.012 |
| PPUBIRD1_2747 | hypothetical protein | -11.50 | 0.020 |
| PPUBIRD1_3231 | hypothetical protein | -13.05 | 0.009 |
| PPUBIRD1_1593 | hypothetical protein | -14.00 | 0.020 |
| PPUBIRD1_3985 | hypothetical protein | -15.32 | 0.014 |
| PPUBIRD1_4148 | hypothetical protein | -16.03 | 0.002 |
| PPUBIRD1_3285 | hypothetical protein | -20.50 | 0.001 |
| PPUBIRD1_3832 | hypothetical protein | -26.50 | 0.003 |
| PPUBIRD1_0753 | hypothetical protein | -28.00 | 0.011 |
| PPUBIRD1_1991 | hypothetical protein | -36.50 | 0.015 |
| PPUBIRD1_0460 | hypothetical protein | -37.50 | 0.004 |
| PPUBIRD1_3014 | hypothetical protein | -39.00 | 0.006 |
| PPUBIRD1_0773 | hypothetical protein | -57.00 | 0.008 |
| PPUBIRD1_0735 | hypothetical protein | -66.50 | 0.007 |
| PPUBIRD1_4723 | hypothetical protein | -75.50 | 0.010 |
| PPUBIRD1_1079 | hypothetical protein | -84.50 | 0.008 |
| PPUBIRD1_0722 | hypothetical protein | -92.00 | 0.002 |
| PPUBIRD1_4306 | hypothetical protein | -94.50 | 0.001 |
| PPUBIRD1_0842 | hypothetical protein | -125.00 | 0.004 |
| **Unclassified proteins** | | | |
| PPUBIRD1_0687 | Fimbrial protein pilin | 35.50 | 0.002 |
| PPUBIRD1_1442 | BkdR | 26.00 | 0.006 |
| PPUBIRD1_4185 | 4-hydroxybenzoate transporter | 20.50 | 0.005 |
| PPUBIRD1_2144 | Flavin reductase domain-containing protein | 10.53 | 0.011 |
| PPUBIRD1_4038 | CspA protein (cold shock protein) | 7.15 | 0.018 |
| PPUBIRD1_2391 | Curlin-associated protein | 4.79 | 0.016 |
| PPUBIRD1_1326 | AAA ATPase | 4.50 | 0.004 |
| PPUBIRD1_2590 | Sugar transferase. putative | 3.76 | 0.004 |
| PPUBIRD1_1752 | UvrC protein | 3.49 | 0.015 |
| PPUBIRD1_3085 | ABC transporter. permease/ATP-binding protein. putative | 2.94 | 0.010 |
| PPUBIRD1_2066 | decarboxylase | 2.82 | 0.019 |
| PPUBIRD1_2581 | Aldehyde dehydrogenase family protein | 2.74 | 0.001 |
| PPUBIRD1_1126 | protein GlpF (glicerol uptake facilitator) | 2.68 | 0.017 |
| PPUBIRD1_3230 | Deoxyribonuclease I | 2.64 | 0.001 |
| PPUBIRD1_2079 | amino acid ABC transporter substrate-binding protein | 2.52 | 0.015 |
| PPUBIRD1_4946 | SerA (D-3-phosphoglycerate dehydrogenase - glicine serine metabolism. methane metabolism) | 2.51 | 0.007 |
| PPUBIRD1_5067 | FAD dependent oxidoreductase | 2.41 | 0.013 |
| PPUBIRD1_2405 | EndA (endonuclease) | 2.34 | 0.008 |
| PPUBIRD1_3233 | FAD dependent oxidoreductase | 2.33 | 0.003 |
| PPUBIRD1_2659 | Methylated-DNA--protein-cysteine methyltransferase | 2.33 | 0.006 |
| PPUBIRD1_2586 | Oxidoreductase. putative | 2.32 | 0.004 |
| PPUBIRD1_3331 | Multi-sensor signal transduction histidine kinase | 2.06 | 0.015 |
| PPUBIRD1_3471 | Putative aminotransferase | 2.05 | 0.002 |
| PPUBIRD1_2640 | Phospho-2-dehydro-3-deoxyheptonate aldolase (tyrosine inhibited-like) | 2.01 | 0.012 |
| PPUBIRD1_4588 | protein MltB (membrane-bound lytic murein transglycosylase B) | -2.11 | 0.011 |
| PPUBIRD1_0024 | Sodium/hydrogen exchanger | -2.12 | 0.005 |
| PPUBIRD1_0148 | Periplasmic solute binding protein | -2.15 | 0.015 |
| PPUBIRD1_3578 | ECF subfamily RNA polymerase sigma-24 factor | -2.16 | 0.015 |
| PPUBIRD1_4149 | Pseudouridine synthase (16S rRNA pseudouridylate synthase A) | -2.20 | 0.007 |
| PPUBIRD1_3333 | Multi-sensor hybrid histidine kinase | -2.22 | 0.003 |
| PPUBIRD1_4581 | Lytic murein transglycosylase | -2.26 | 0.005 |
| PPUBIRD1_4440 | D-lactate dehydrogenase (pyruvate metabolism) | -2.26 | 0.017 |
| PPUBIRD1_2131 | Permease for cytosine/purine. uracil. thiamine. allantoin | -2.42 | 0.007 |
| PPUBIRD1_2810 | Mqo3 (malate:quinone oxidoreductase - pyruvate metabolism) | -2.54 | 0.013 |
| PPUBIRD1_2746 | Prophage PSPPH02. adenine modification methytransferase | -2.58 | 0.010 |
| PPUBIRD1_3247 | aminotransferase. class V (thiamine metabolism) | -2.71 | 0.014 |
| PPUBIRD1_1468 | protein FliS (flagellar protein FliS) | -2.76 | 0.009 |
| PPUBIRD1_0882 | endoribonuclease L-PSP | -2.78 | 0.002 |
| PPUBIRD1_0649 | Paraquat-inducible protein A | -2.80 | 0.013 |
| PPUBIRD1_2766 | portal protein | -2.97 | 0.003 |
| PPUBIRD1_2868 | Pyridine nucleotide-disulfide oxidoreductase family protein | -3.00 | 0.004 |
| PPUBIRD1_3915 | RdgC (DNA recombination-dependent growth factor C) | -3.03 | 0.003 |
| PPUBIRD1_3541 | Pseudouridine synthase | -3.09 | 0.001 |
| PPUBIRD1_2502 | Protein maoC | -3.40 | 0.017 |
| PPUBIRD1_0186 | Nicotinamide nucleotide transhydrogenase subunit alpha 1 | -3.44 | 0.018 |
| PPUBIRD1_0329 | Ricin B lectin | -3.71 | 0.012 |
| PPUBIRD1_4207 | AmpG-related permease (b-lactamase related) | -4.02 | 0.018 |
| PPUBIRD1_2777 | Phage integrase family protein | -4.11 | 0.015 |
| PPUBIRD1_2835 | Acyl-homoserine lactone acylase pvdQ | -4.34 | 0.012 |
| PPUBIRD1_4531 | Site-specific recombinase. phage integrase family domain protein | -4.70 | 0.011 |
| PPUBIRD1_0291 | Integral membrane sensor signal transduction histidine kinase | -4.94 | 0.008 |
| PPUBIRD1_1458 | protein FlgH (Flagellar basal body L-ring protein) | -5.00 | 0.019 |
| PPUBIRD1_4726 | Glycosyl transferase. putative | -5.07 | 0.003 |
| PPUBIRD1_3540 | methyl-accepting chemotaxis sensory transducer | -5.18 | 0.014 |
| PPUBIRD1_3803 | ABC transporter | -5.31 | 0.013 |
| PPUBIRD1_3796 | Alcohol dehydrogenase. zinc-containing (quinone reductase) | -5.97 | 0.003 |
| PPUBIRD1_0516 | protein RpoA (DNA-directed RNA polymerase subunit alpha) | -6.00 | 0.002 |
| PPUBIRD1_1990 | Putative phage repressor | -8.81 | 0.001 |
| PPUBIRD1_2780 | IstB domain-containing protein ATP-binding protein | -9.13 | 0.016 |
| PPUBIRD1_4532 | phage integrase family site-specific recombinase | -9.78 | 0.015 |
| PPUBIRD1_1551 | Major facilitator transporter | -10.50 | 0.020 |
| PPUBIRD1_1345 | PhaJ1 (MaoC dehydratase) | -10.55 | 0.019 |
| PPUBIRD1_2765 | Peptidase S14 ClpP | -10.91 | 0.005 |
| PPUBIRD1_2764 | Major head protein | -11.59 | 0.004 |
| PPUBIRD1_1845 | NAD-dependent epimerase/dehydratase | -11.81 | 0.010 |
| PPUBIRD1_4236 | Uroporphyrin-III C/tetrapyrrole methyltransferase | -12.48 | 0.001 |
| PPUBIRD1_4312 | leucine dehydrogenase (Valine. leucine and isoleucine degradation) | -12.82 | 0.006 |
| PPUBIRD1_0286 | HAD family hydrolase | -15.33 | 0.010 |
| PPUBIRD1_4511 | Major facilitator family transporter | -21.50 | 0.001 |
| PPUBIRD1_2825 | GABA permease (Gamma-aminobutyrate permease) | -23.50 | 0.011 |
| PPUBIRD1_1395 | Spy-related protein (bacterial toxin) | -34.50 | 0.000 |
| PPUBIRD1_0926 | FAD dependent oxidoreductase | -40.00 | 0.001 |
| PPUBIRD1_4889 | nucleoside-triphosphatase (purine thiamine metabolism) | -42.00 | 0.014 |
| PPUBIRD1_0002 | transglycosylase | -43.50 | 0.016 |
| PPUBIRD1_4508 | Amino acid permease-associated region | -44.00 | 0.001 |
| PPUBIRD1_1450 | protein CheR | -79.00 | 0.013 |
| PPUBIRD1_3867 | Carbon storage regulator. CsrA | -235.00 | 0.003 |
| PPUBIRD1_2772 | Host specificity protein J | -263.00 | 0.003 |

| **Shock butanol 0.5%** | |  |  |
| --- | --- | --- | --- |
| **Synonym** | **Product** | **Fold change** | **p-value** |
| **TCA cycle and related proteins** | | | |
| PPUBIRD1_2165 | Gluconate 2-dehydrogenase acceptor subunit | -2.16 | 0.012 |
| PPUBIRD1_0284 | protein FdhD (formate dehydrogenase accessory protein) | -2.38 | 0.014 |
| PPUBIRD1_0948 | Hydro-lyase. Fe-S type. tartrate/fumarate subfamily. alpha subunit (citrate cycle) | -2.41 | 0.009 |
| PPUBIRD1_4974 | PotG (ABC-type spermidine/putrescine transport systems. ATPase components - glutathione arginine metabolism related) | -3.03 | 0.019 |
| PPUBIRD1_1071 | DNA-binding transcriptional regulator HexR | -3.79 | 0.004 |
| PPUBIRD1_4844 | protein Pgm | -4.08 | 0.008 |
| PPUBIRD1_1070 | aldose 1-epimerase (glycolisis) | -13.92 | 0.014 |
| PPUBIRD1_1777 | Gnd (6-phosphogluconate dehydrogenase-like protein) | -14 | 0.000 |
| PPUBIRD1_4980 | ArgA (arginine and proline metabolism) | -15 | 0.010 |
| PPUBIRD1_2490 | protein PaaC (3-hydroxyacyl-CoA dehydrogenase- phenylalanine metabolism) | -20 | 0.020 |
| PPUBIRD1_3076 | Major facilitator family transporter (Sugar phosphate permease) | -23 | 0.000 |
| PPUBIRD1_2373 | Carbohydrate kinase | -28 | 0.001 |
| PPUBIRD1_0319 | protein HisH (Glutamine amidotransferase ) | -29 | 0.001 |
| PPUBIRD1_3438 | FadD protein (glycolisis metabolism) | -30 | 0.002 |
| PPUBIRD1_2404 | gluconate 2-dehydrogenase | -31 | 0.000 |
| PPUBIRD1_2495 | PaaH (phenylacetate-CoA oxygenase subunit PaaB) | -33 | 0.017 |
| PPUBIRD1_3791 | glutathione S-transferase | -77.75 | 0.001 |
| PPUBIRD1_2709 | glutaredoxin | -80 | 0.000 |
| PPUBIRD1_4379 | protein IlvH (butanoate metabolism) | -108 | 0.001 |
| **Efflux pumps and resistance proteins** | | | |
| PPUBIRD1_1892 | TetR family transcriptional regulator | -19 | 0.003 |
| PPUBIRD1_2078 | TetR family transcriptional regulator | -31 | 0.005 |
| PPUBIRD1_4265 | Carboxylesterase (drug metabolism) | -76 | 0.005 |
| **Lipid metabolism** | | | |
| PPUBIRD1_1334 | Putative lipoprotein | 45 | 0.008 |
| PPUBIRD1_1788 | lipocalin family protein | 2.78 | 0.019 |
| PPUBIRD1_4952 | Lysophospholipase-like protein | -2.14 | 0.004 |
| PPUBIRD1_4596 | PAP2 family protein/DedA family protein | -2.29 | 0.007 |
| PPUBIRD1_0429 | Glycerol-3-phosphate acyltransferase | -4.22 | 0.013 |
| PPUBIRD1_3766 | Enoyl-CoA hydratase | -12.93 | 0.012 |
| PPUBIRD1_3437 | FadB2 | -23.00 | 0.015 |
| PPUBIRD1_2030 | Enoyl-CoA hydratase/isomerase | -30.00 | 0.011 |
| PPUBIRD1_0655 | protein LspA (lipoprotein signal peptidase) | -37.00 | 0.019 |
| PPUBIRD1_1261 | OprL (peptidoglycan-associated outer membrane lipoprotein) | -59.00 | 0.001 |
| PPUBIRD1_0591 | Ethanolamine ammonia-lyase light chain (Glycerophospholipid metabolism) | -128.0 | 0.015 |
| **Ferric related proteins** | | | |
| PPUBIRD1_0912 | TonB-dependent siderophore receptor | -6.65 | 0.018 |
| PPUBIRD1_0917 | Anti-FecI sigma factor. FecR | -19 | 0.014 |
| **Energy production** | | | |
| PPUBIRD1_0859 | CyoD protein (cytochrome or ubiquinol subunit IV oxidase) | 102 | 0.002 |
| PPUBIRD1_4863 | HemE protein | -2.46 | 0.015 |
| PPUBIRD1_0415 | PqqB (pyrroloquinoline quinone biosynthesis protein PqqB) | -2.78 | 0.002 |
| PPUBIRD1_2184 | Qor (quinone oxidoreductase. NADPH-dependent) | -14 | 0.000 |
| PPUBIRD1_4992 | UbiF (Ubiquinone biosynthesis hydroxylase) | -16 | 0.018 |
| PPUBIRD1_1605 | CcoO_2 (cytochrome c subunit) | -40 | 0.005 |
| **Cell division** | | | |
| PPUBIRD1_4222 | protein FtsA (cell division protein FtsA) | -2.50 | 0.007 |
| PPUBIRD1_4233 | cell division protein FtsL | -20 | 0.005 |
| **Transcriptional regulators** | | | |
| PPUBIRD1_2250 | GntR family transcriptional regulator | 2.02 | 0.014 |
| PPUBIRD1_2294 | Sigma54 specific transcriptional regulator. Fis family | -2.26 | 0.013 |
| PPUBIRD1_4445 | LysR family transcriptional regulator (DNA-binding transcriptional activator GcvA) | -4.94 | 0.005 |
| PPUBIRD1_0952 | ColR | -12.6 | 0.015 |
| PPUBIRD1_1488 | Two component. sigma54 specific. Fis family transcriptional regulator | -14 | 0.000 |
| PPUBIRD1_3470 | Cro/CI family transcriptional regulator | -19 | 0.001 |
| PPUBIRD1_1022 | GntR family transcriptional regulator | -19 | 0.018 |
| PPUBIRD1_3934 | MarR family transcriptional regulator | -49 | 0.003 |
| PPUBIRD1_4604 | Fis | -74 | 0.001 |
| PPUBIRD1_5062 | Cro/CI family transcriptional regulator | -98 | 0.013 |
| PPUBIRD1_1433 | AlgZ protein (alginate production) | -228 | 0.001 |
| **tRNA related proteins** | | | |
| PPUBIRD1_0473 | TyrS (Tyrosyl-tRNA synthetase) | -25 | 0.000 |
| PPUBIRD1_4350 | protein TrmA (tRNA (uracil-5-)-methyltransferase) | -36 | 0.002 |
| PPUBIRD1_t0033 | Ser tRNA | -255 | 0.000 |
| **Hypothetical proteins** | | | |
| PPUBIRD1_1249 | hypothetical protein | 27.5 | 0.002 |
| PPUBIRD1_3923 | hypothetical protein | 4.71 | 0.003 |
| PPUBIRD1_2435 | hypothetical protein | 4.49 | 0.017 |
| PPUBIRD1_1733 | hypothetical protein | 4.08 | 0.012 |
| PPUBIRD1_0302 | hypothetical protein | 3.31 | 0.019 |
| PPUBIRD1_2678 | hypothetical protein | 2.92 | 0.006 |
| PPUBIRD1_3980 | hypothetical protein | -2.07 | 0.009 |
| PPUBIRD1_3461 | hypothetical protein | -2.14 | 0.003 |
| PPUBIRD1_3983 | hypothetical protein | -2.17 | 0.016 |
| PPUBIRD1_0832 | hypothetical protein | -2.32 | 0.013 |
| PPUBIRD1_0234 | hypothetical protein | -2.47 | 0.006 |
| PPUBIRD1_2371 | hypothetical protein | -2.93 | 0.004 |
| PPUBIRD1_3156 | hypothetical protein | -2.97 | 0.019 |
| PPUBIRD1_3231 | hypothetical protein | -3.22 | 0.012 |
| PPUBIRD1_1388 | hypothetical protein | -4.05 | 0.001 |
| PPUBIRD1_4703 | hypothetical protein | -4.24 | 0.003 |
| PPUBIRD1_4757 | hypothetical protein | -4.32 | 0.014 |
| PPUBIRD1_5087 | hypothetical protein | -4.60 | 0.014 |
| PPUBIRD1_4662 | hypothetical protein | -7.04 | 0.000 |
| PPUBIRD1_4148 | hypothetical protein | -7.43 | 0.002 |
| PPUBIRD1_4923 | hypothetical protein | -14 | 0.009 |
| PPUBIRD1_3635 | hypothetical protein | -15 | 0.008 |
| PPUBIRD1_1545 | hypothetical protein | -17 | 0.004 |
| PPUBIRD1_3013 | hypothetical protein | -21 | 0.004 |
| PPUBIRD1_0753 | hypothetical protein | -25 | 0.011 |
| PPUBIRD1_2602 | hypothetical protein | -29 | 0.014 |
| PPUBIRD1_1219 | hypothetical protein | -30.3 | 0.005 |
| PPUBIRD1_4622 | hypothetical protein | -32 | 0.007 |
| PPUBIRD1_0988 | hypothetical protein | -33 | 0.018 |
| PPUBIRD1_4050 | hypothetical protein | -33.8 | 0.002 |
| PPUBIRD1_1165 | hypothetical protein | -38.3 | 0.013 |
| PPUBIRD1_0453 | hypothetical protein | -42 | 0.004 |
| PPUBIRD1_3739 | hypothetical protein | -42 | 0.018 |
| PPUBIRD1_2983 | hypothetical protein | -43 | 0.013 |
| PPUBIRD1_4551 | hypothetical protein | -44 | 0.016 |
| PPUBIRD1_4939 | hypothetical protein | -45 | 0.001 |
| PPUBIRD1_1989 | hypothetical protein | -48 | 0.002 |
| PPUBIRD1_2823 | hypothetical protein | -57 | 0.011 |
| PPUBIRD1_4688 | hypothetical protein | -63 | 0.001 |
| PPUBIRD1_0460 | hypothetical protein | -75 | 0.005 |
| PPUBIRD1_1732 | hypothetical protein | -75 | 0.007 |
| PPUBIRD1_1079 | hypothetical protein | -77 | 0.008 |
| PPUBIRD1_4161 | hypothetical protein | -81 | 0.000 |
| PPUBIRD1_3298 | hypothetical protein | -84 | 0.001 |
| PPUBIRD1_0629 | hypothetical protein | -84 | 0.007 |
| PPUBIRD1_0899 | hypothetical protein | -90 | 0.006 |
| PPUBIRD1_3202 | hypothetical protein | -93 | 0.000 |
| PPUBIRD1_0679 | hypothetical protein | -98 | 0.003 |
| PPUBIRD1_4306 | hypothetical protein | -98 | 0.001 |
| PPUBIRD1_2019 | hypothetical protein | -105 | 0.001 |
| PPUBIRD1_4701 | hypothetical protein | -106 | 0.009 |
| PPUBIRD1_1314 | hypothetical protein | -117 | 0.000 |
| PPUBIRD1_2761 | hypothetical protein | -154 | 0.000 |
| PPUBIRD1_2933 | hypothetical protein | -171 | 0.008 |
| PPUBIRD1_3798 | hypothetical protein | -269 | 0.000 |
| **Unclassified proteins** | | | |
| PPUBIRD1_3394 | sugar ABC transporter ATP-binding protein | 14.50 | 0.011 |
| PPUBIRD1_4811 | Polar amino acid ABC transporter. inner membrane subunit | 9.86 | 0.019 |
| PPUBIRD1_1505 | protein FliR (Flagellar biosynthesis pathway. component FliR) | 4.14 | 0.011 |
| PPUBIRD1_0256 | TauD (taurine dioxygenase) | 2.98 | 0.018 |
| PPUBIRD1_1827 | short-chain dehydrogenase | 2.74 | 0.020 |
| PPUBIRD1_2998 | Beta-lactamase domain protein | 2.38 | 0.011 |
| PPUBIRD1_3922 | Integral membrane sensor hybrid histidine kinase | -2.15 | 0.001 |
| PPUBIRD1_4581 | Lytic murein transglycosylase | -2.24 | 0.006 |
| PPUBIRD1_4907 | Alpha/beta fold family hydrolase | -2.33 | 0.019 |
| PPUBIRD1_4440 | D-lactate dehydrogenase (pyruvate metabolism) | -2.33 | 0.014 |
| PPUBIRD1_4339 | uracil-xanthine permease | -2.64 | 0.015 |
| PPUBIRD1_1038 | GcvP (glicine threonine metabolism) | -2.66 | 0.009 |
| PPUBIRD1_3256 | Alcohol dehydrogenase (Zn-dependent alcohol dehydrogenases) | -2.82 | 0.007 |
| PPUBIRD1_2126 | Phage integrase family protein | -3 | 0.020 |
| PPUBIRD1_4149 | Pseudouridine synthase | -3.21 | 0.007 |
| PPUBIRD1_2407 | Surface antigen (D15) (outer membrane protein assembly factor) | -3.34 | 0.012 |
| PPUBIRD1_3425 | Putative monovalent cation/H+ antiporter subunit C | -3.35 | 0.008 |
| PPUBIRD1_1548 | mechanosensitive ion channel protein MscS | -3.37 | 0.002 |
| PPUBIRD1_3520 | Universal stress protein | -3.79 | 0.002 |
| PPUBIRD1_3333 | Multi-sensor hybrid histidine kinase | -3.81 | 0.010 |
| PPUBIRD1_1155 | ATP-dependent DNA ligase | -4.05 | 0.002 |
| PPUBIRD1_4236 | Uroporphyrin-III C/tetrapyrrole methyltransferase | -4.07 | 0.020 |
| PPUBIRD1_2135 | GAF sensor hybrid histidine kinase | -5.18 | 0.012 |
| PPUBIRD1_3977 | Protein sprT | -5.33 | 0.003 |
| PPUBIRD1_2153 | ABC-type nitrate/sulfonate/bicarbonate transport systems periplasmic components-like protein | -8 | 0.003 |
| PPUBIRD1_0517 | protein RplQ (ribosomal protein L17) | -10.53 | 0.019 |
| PPUBIRD1_3558 | Nitrate-binding protein NasS. putative (ABC-type nitrate/sulfonate/bicarbonate transport systems. periplasmic components) | -10.59 | 0.020 |
| PPUBIRD1_4565 | ThiD (thiamine metabolism) | -11.00 | 0.016 |
| PPUBIRD1_4846 | Carboxyl-terminal protease | -11.44 | 0.018 |
| PPUBIRD1_2685 | AroE_2 (shikimate - phenylalanine. tryptophan metabolism) | -12.00 | 0.020 |
| PPUBIRD1_1551 | Major facilitator transporter | -12.00 | 0.020 |
| PPUBIRD1_4202 | GroES protein (chaperonine - heat shock protein) | -13.86 | 0.006 |
| PPUBIRD1_3560 | Nitrite transporter | -16 | 0.020 |
| PPUBIRD1_2334 | Acyl-CoA synthetase | -18 | 0.008 |
| PPUBIRD1_2402 | Ribokinase-like domain-containing protein (pentose phosphate) | -20.00 | 0.020 |
| PPUBIRD1_1214 | DctP (TRAP-type C4-dicarboxylate transport system) | -21.00 | 0.020 |
| PPUBIRD1_2484 | Universal stress protein | -21.00 | 0.004 |
| PPUBIRD1_3046 | Response regulator receiver/ANTAR domain-containing protein | -22.00 | 0.002 |
| PPUBIRD1_2585 | Periplasmic polyamine-binding protein. putative (putrescine transporter subunit: periplasmic-binding component of ABC superfamily) | -24 | 0.000 |
| PPUBIRD1_2412 | Major facilitator family transporter | -26.00 | 0.011 |
| PPUBIRD1_1799 | HflD-like high frequency lysogenization protein | -27.00 | 0.006 |
| PPUBIRD1_2053 | CatA (toluene. benzoates. hexanes degradation) | -28.00 | 0.001 |
| PPUBIRD1_4096 | protein RimM (16S rRNA-processing protein RimM) | -28.00 | 0.001 |
| PPUBIRD1_1259 | Protein TolA | -28.00 | 0.009 |
| PPUBIRD1_4588 | protein MltB (membrane-bound lytic murein transglycosylase B) | -29.00 | 0.000 |
| PPUBIRD1_3550 | Deoxyguanosinetriphosphate triphosphohydrolase-like protein | -30.00 | 0.017 |
| PPUBIRD1_5078 | RadC (DNA repair protein radc) | -33.25 | 0.001 |
| PPUBIRD1_4726 | Glycosyl transferase. putative | -34.00 | 0.002 |
| PPUBIRD1_1395 | Spy-related protein (bacterial toxin) | -35.00 | 0.000 |
| PPUBIRD1_4281 | AlgI protein | -37.00 | 0.003 |
| PPUBIRD1_3204 | Integrase family protein | -37.00 | 0.001 |
| PPUBIRD1_2848 | Catalase domain protein | -38.00 | 0.000 |
| PPUBIRD1_0693 | ISPsy5. Orf1 | -41.00 | 0.003 |
| PPUBIRD1_2929 | UspA domain-containing protein | -41 | 0.000 |
| PPUBIRD1_4125 | LepA protein (GTP-binding protein LepA) | -44.00 | 0.010 |
| PPUBIRD1_4068 | Putative CheW protein | -44.00 | 0.000 |
| PPUBIRD1_3031 | Helix-turn-helix domain-containing protein | -46.00 | 0.005 |
| PPUBIRD1_4719 | O-antigen polymerase | -48 | 0.013 |
| PPUBIRD1_3620 | ATPase | -52.00 | 0.014 |
| PPUBIRD1_0128 | CynT (nitrogen metabolism) | -53.00 | 0.019 |
| PPUBIRD1_0606 | ATP-NAD/AcoX kinase | -57.00 | 0.001 |
| PPUBIRD1_1180 | Membrane protein-like protein | -64.00 | 0.000 |
| PPUBIRD1_1458 | protein FlgH (Flagellar basal body L-ring protein) | -65.00 | 0.002 |
| PPUBIRD1_0506 | protein RplF (50S ribosomal protein L6) | -78.00 | 0.009 |
| PPUBIRD1_0389 | DNA polymerase III subunit epsilon | -83.00 | 0.018 |
| PPUBIRD1_0355 | SoxD (glicine serine threonine metabolism) | -96.00 | 0.003 |
| PPUBIRD1_0439 | KsgA (16S ribosomal RNA methyltransferase KsgA/Dim1 family protein) | -134.0 | 0.013 |
| PPUBIRD1_0465 | Histidine triad (HIT) protein | -157.0 | 0.001 |
| PPUBIRD1_3867 | Carbon storage regulator. CsrA | -247.0 | 0.003 |
