# Supplementary figures and images for: Understanding butanol tolerance and assimilation in P seudomonas putida BIRD‐1: an integrated omics approach
Source: Microb Biotechnol. 2016 Jan 6;9(1):100–15. doi: 10.1111/1751-7915.12328 (PMC4720416; doi:10.1111/1751-7915.12328)

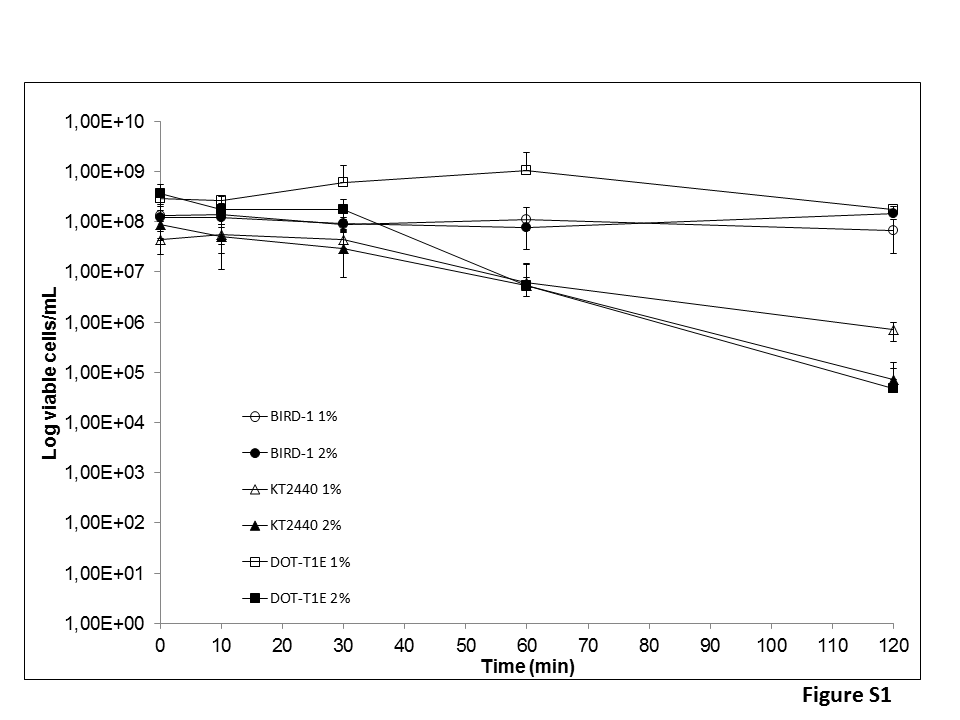

Supplement: Supplementary file 1 — Fig. S1. Cell death kinetics after a butanol shock of BIRD‐1, KT2440 and DOT‐T1E. Killing kinetics of P. putida strains upon exposure to different butanol concentrations. The strains were grown to reach the exponential phase (turbidity of 0.85 at 660 nm). At t = 0 the culture was divided into two aliquots, to which 1% or 2% (v/v) butanol was added. At the indicated times, the number of viable cells were estimated by plating dilutions on LB. Fig. S2. ppGpp response model. ppGpp accumulation is mediated by the SpoT protein. In the genome, spoT is located downstream of rpoZ, which is the omega subunit of RNA polymerase. Table S1. Doubling time of P. putida BIRD‐1, KT2440 and DOT‐T1E growing on different media. Doubling times (G) and lag phases (lag) are indicated. Table S2. Mutant library characteristics and phenotypes. Mutants in a mutant library, insertion points of the sequences obtained and phenotype (A, assimilation, T, tolerance and A&T, assimilation and tolerance). Table S3. Venn Diagram specification. Butanol as sole carbon source, Shock and glucose butanol grown cells. Each transcript found in common in the diagram is categorized. Table S4. Transcriptomics results. Table obtained after comparison of the all the conditions versus the control (glucose grown cells). Table S5. Peptides whole cell proteome detected by MS/MS List of redundant peptides obtained from whole cell proteome of the three biological replicates of the control (C), butanol grown cells (B), glucose plus butanol grown cells (GB) and cells after a butanol shock (S). Table S6. Pattern Lab analysis of whole cell proteome. List of proteins from whole cells of P. putida BIRD‐1 validated with at least two different peptides. Table S7. Peptides membrane proteome detected by MS/MS. List of redundant peptides obtained from membrane proteins of the three biological replicates of the control (C), butanol grown cells (B), glucose plus butanol grown cells (GB) and cells after a butanol shock (S). Table S8. [file MBT2-9-100-s001.zip › Figure S1.TIF]

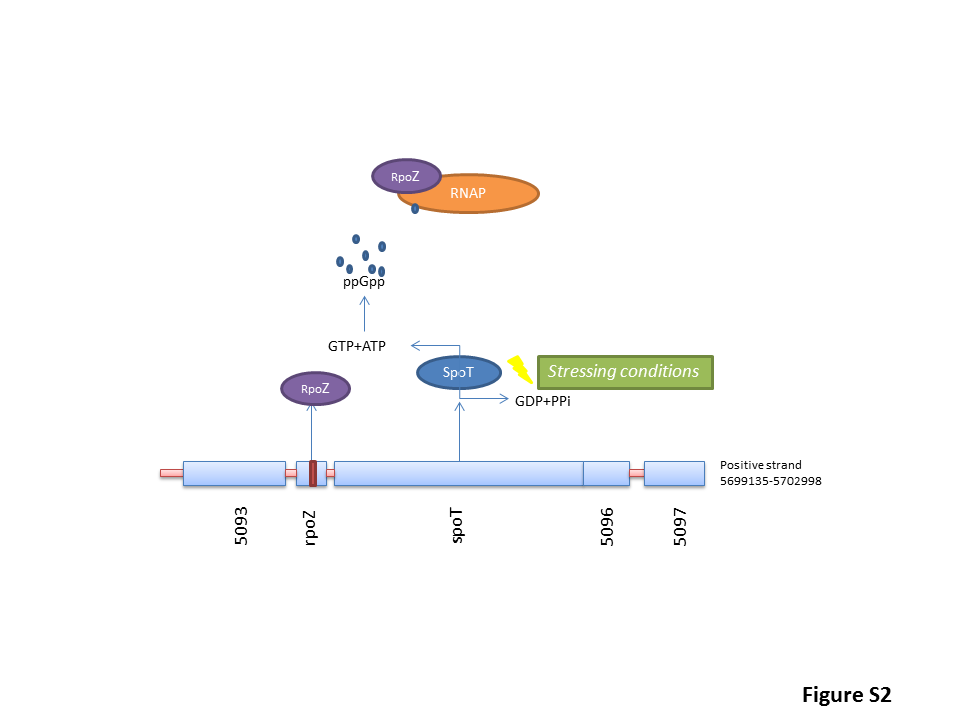

Supplement: Supplementary file 1 — Fig. S1. Cell death kinetics after a butanol shock of BIRD‐1, KT2440 and DOT‐T1E. Killing kinetics of P. putida strains upon exposure to different butanol concentrations. The strains were grown to reach the exponential phase (turbidity of 0.85 at 660 nm). At t = 0 the culture was divided into two aliquots, to which 1% or 2% (v/v) butanol was added. At the indicated times, the number of viable cells were estimated by plating dilutions on LB. Fig. S2. ppGpp response model. ppGpp accumulation is mediated by the SpoT protein. In the genome, spoT is located downstream of rpoZ, which is the omega subunit of RNA polymerase. Table S1. Doubling time of P. putida BIRD‐1, KT2440 and DOT‐T1E growing on different media. Doubling times (G) and lag phases (lag) are indicated. Table S2. Mutant library characteristics and phenotypes. Mutants in a mutant library, insertion points of the sequences obtained and phenotype (A, assimilation, T, tolerance and A&T, assimilation and tolerance). Table S3. Venn Diagram specification. Butanol as sole carbon source, Shock and glucose butanol grown cells. Each transcript found in common in the diagram is categorized. Table S4. Transcriptomics results. Table obtained after comparison of the all the conditions versus the control (glucose grown cells). Table S5. Peptides whole cell proteome detected by MS/MS List of redundant peptides obtained from whole cell proteome of the three biological replicates of the control (C), butanol grown cells (B), glucose plus butanol grown cells (GB) and cells after a butanol shock (S). Table S6. Pattern Lab analysis of whole cell proteome. List of proteins from whole cells of P. putida BIRD‐1 validated with at least two different peptides. Table S7. Peptides membrane proteome detected by MS/MS. List of redundant peptides obtained from membrane proteins of the three biological replicates of the control (C), butanol grown cells (B), glucose plus butanol grown cells (GB) and cells after a butanol shock (S). Table S8. [file MBT2-9-100-s001.zip › Figure S2.TIF]
